# Host-associated microbiomes are predicted by immune system complexity and climate

**DOI:** 10.1186/s13059-019-1908-8

**Published:** 2020-02-03

**Authors:** Douglas C. Woodhams, Molly C. Bletz, C. Guilherme Becker, Hayden A. Bender, Daniel Buitrago-Rosas, Hannah Diebboll, Roger Huynh, Patrick J. Kearns, Jordan Kueneman, Emmi Kurosawa, Brandon C. LaBumbard, Casandra Lyons, Kerry McNally, Klaus Schliep, Nachiket Shankar, Amanda G. Tokash-Peters, Miguel Vences, Ross Whetstone

**Affiliations:** 1 Department of Biology, University of Massachusetts Boston, Boston, MA 02125 USA; 2 Smithsonian Tropical Research Institute, Roosevelt Ave. Tupper Building – 401, 0843-03092 Panamá, Panama; 3 Department of Biological Sciences, The University of Alabama, Tuscaloosa, AL 35487 USA; 4 School for the Environment, University of Massachusetts, Boston, MA 02125 USA; 5 Animal Health Department, New England Aquarium, Boston, MA 02110 USA; 6 Center of Excellence in Biodiversity and Natural Resource Management, University of Rwanda, RN1, Butare, Rwanda; 7 Zoological Institute, Braunschweig University of Technology, Mendelssohnstr. 4, 38106 Braunschweig, Germany

**Keywords:** Biodiversity, Gut microbiome, Microbial ecology, Skin microbiome, Symbiosis, *Wolbachia*

## Abstract

**Background:**

Host-associated microbiomes, the microorganisms occurring inside and on host surfaces, influence evolutionary, immunological, and ecological processes. Interactions between host and microbiome affect metabolism and contribute to host adaptation to changing environments. Meta-analyses of host-associated bacterial communities have the potential to elucidate global-scale patterns of microbial community structure and function. It is possible that host surface-associated (external) microbiomes respond more strongly to variations in environmental factors, whereas internal microbiomes are more tightly linked to host factors.

**Results:**

Here, we use the dataset from the Earth Microbiome Project and accumulate data from 50 additional studies totaling 654 host species and over 15,000 samples to examine global-scale patterns of bacterial diversity and function. We analyze microbiomes from non-captive hosts sampled from natural habitats and find patterns with bioclimate and geophysical factors, as well as land use, host phylogeny, and trophic level/diet. Specifically, external microbiomes are best explained by variations in mean daily temperature range and precipitation seasonality. In contrast, internal microbiomes are best explained by host factors such as phylogeny/immune complexity and trophic level/diet, plus climate.

**Conclusions:**

Internal microbiomes are predominantly associated with top-down effects, while climatic factors are stronger determinants of microbiomes on host external surfaces. Host immunity may act on microbiome diversity through top-down regulation analogous to predators in non-microbial ecosystems. Noting gaps in geographic and host sampling, this combined dataset represents a global baseline available for interrogation by future microbial ecology studies.

## Background

“A memory-based immune system may have evolved in vertebrates because of the need to recognize and manage complex communities of beneficial microbes.”—McFall-Ngai 2007 [1].

While global patterns of diversity and biogeography have been extensively studied in animals and plants, they are much less understood in microbes. In soil microbiomes, pH has been found to be a strong driver of ecosystem type [2], while in another study, environmental microbiomes were primarily driven by salinity [3]. The recent Earth Microbiome Project found that host microbiomes were distinct from environmental microbiomes, and for hosts, ecosystem type was an important driver. Microbiomes of plants and animals differed strongly, and host surfaces were different from digestive-associated microbiomes [4]. A large study examining skin surface microbiomes across 205 amphibian species found strong correlations with bioclimatic factors [5], while digestive microbiomes of mammals were influenced by diet and gut morphology, and indeed distinct from environmental microbiomes [6]. A substantial role of biotic interactions in shaping microbial communities was also obvious from a strong bacterial-fungal antagonism revealed by global patterns in topsoil and ocean microbiomes [7], a pattern also revealed on hosts [8–11].

**Fig. 1:**
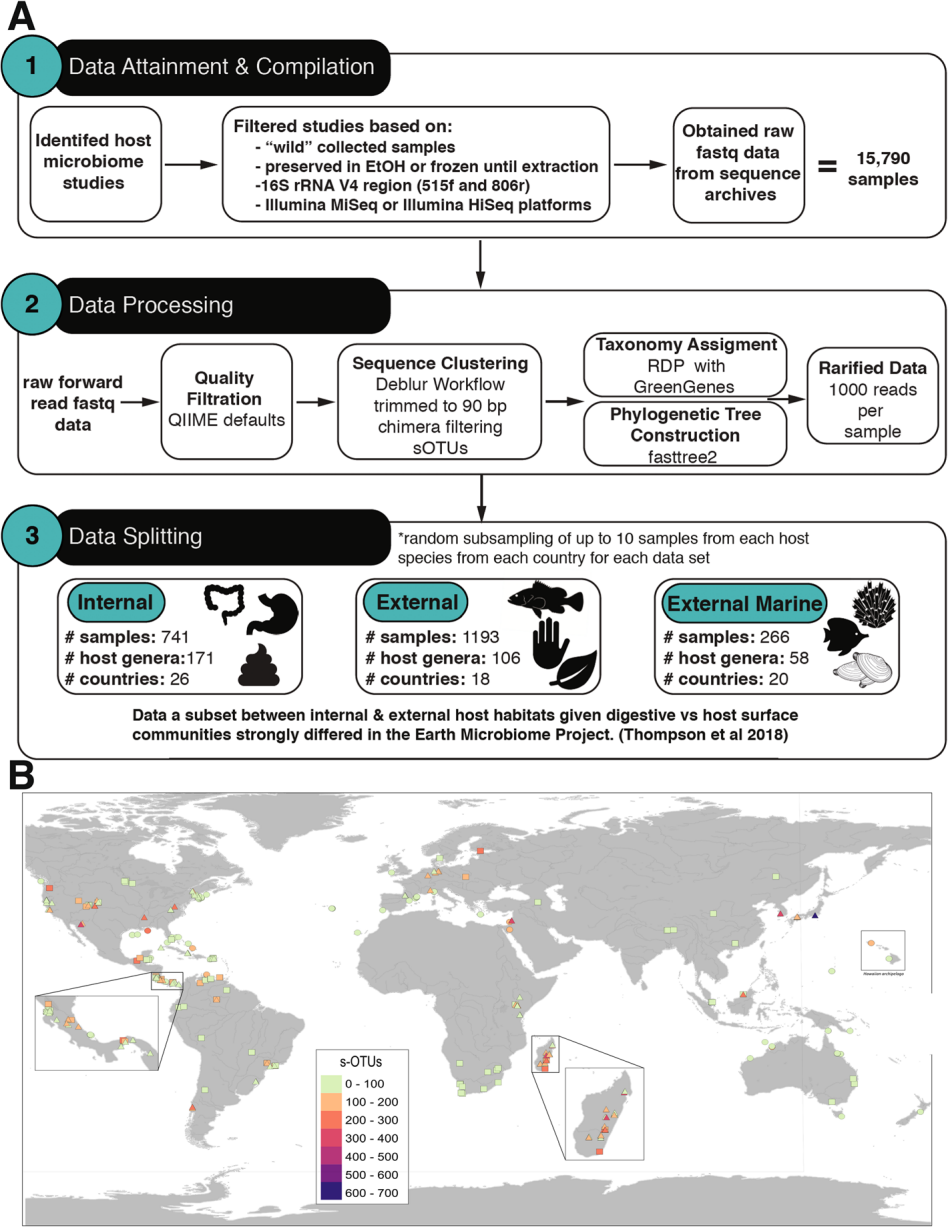
Method schematic and geographic distribution of samples analyzed. **a** Method schematic for data attainment and compilation, data processing, and data splitting into three distinct subsets for subsequent analyses. **b** Map of the coverage of samples included in this study. Three types of host microbiome samples are represented: internal (squares), external (triangles), and marine external (circles). Sampling points are color-scaled by sub-Operational Taxonomic Unit (sOTU) richness. Areas with small territory size (such as Central America and Hawaiian Archipelago) and many sampling points with different types of samples (Madagascar) are shown zoomed-in in separate boxes. Map created with QGIS (Quantum GIS Development Team 2013) using a base global map from Natural Earth (naturalearthdata.com) with all geographic coordinates standardized to decimal degrees

These studies are foundational for understanding large-scale microbial ecology patterns. Here, we use data of host-associated bacterial communities from the Earth Microbiome Project and 50 additional studies that meet our criteria for inclusion to produce a large dataset for analysis through a standardized pipeline (Fig. 1a). We examine 654 non-captive host species including plants, and invertebrate and vertebrate animals and perform separate analyses for digestive-associated (internal) and surface-associated (external) microbiomes from marine or terrestrial/aquatic habitats. With an increasing diversity of hosts examined for symbiosis with microbes, mechanisms driving these host-microbe interactions will become clear [12]. We hypothesized that external host-associated microbiomes would most strongly correlate with global bioclimate or other abiotic factors, while internal microbiomes would be more strongly associated with host factors such as phylogeny, and trophic level or diet.

Diverse microbial communities can assemble and coevolve with hosts and contribute important or even essential functions for host development, physiology, and health [13, 14]. Detrimental alterations in the host’s microbiome, or "dysbiosis", can lead to disease; thus, baseline descriptions of functional microbiomes are essential [15, 16]. In the future, personalized medicine may utilize the microbiome for biomarkers for conditions of disease or health [17], and disease progression may be more readily predicted and described by changes in microbiota than by clinical symptoms or the presence of certain pathogenic agents [18, 19]. Functional characterization may be more meaningful than identity in microbial community profiles due to functional redundancy among microbes [20, 21]. However, characterizing core (prevalent among individuals) microbial communities may also be utilized to develop screening tools for host health or to understand eco-evolutionary dynamics [22, 23]. Here, we hypothesized that internal microbiomes may represent an extension of host phenotype; rather than functioning to reduce microbial diversity, complexity in host immune systems may be correlated with microbiome diversity across taxa. Furthermore, host microbiomes may be more strongly differentiated by predicted community function than by community composition given a multitude of species with overlapping functional capacities.

**Fig. 2:**
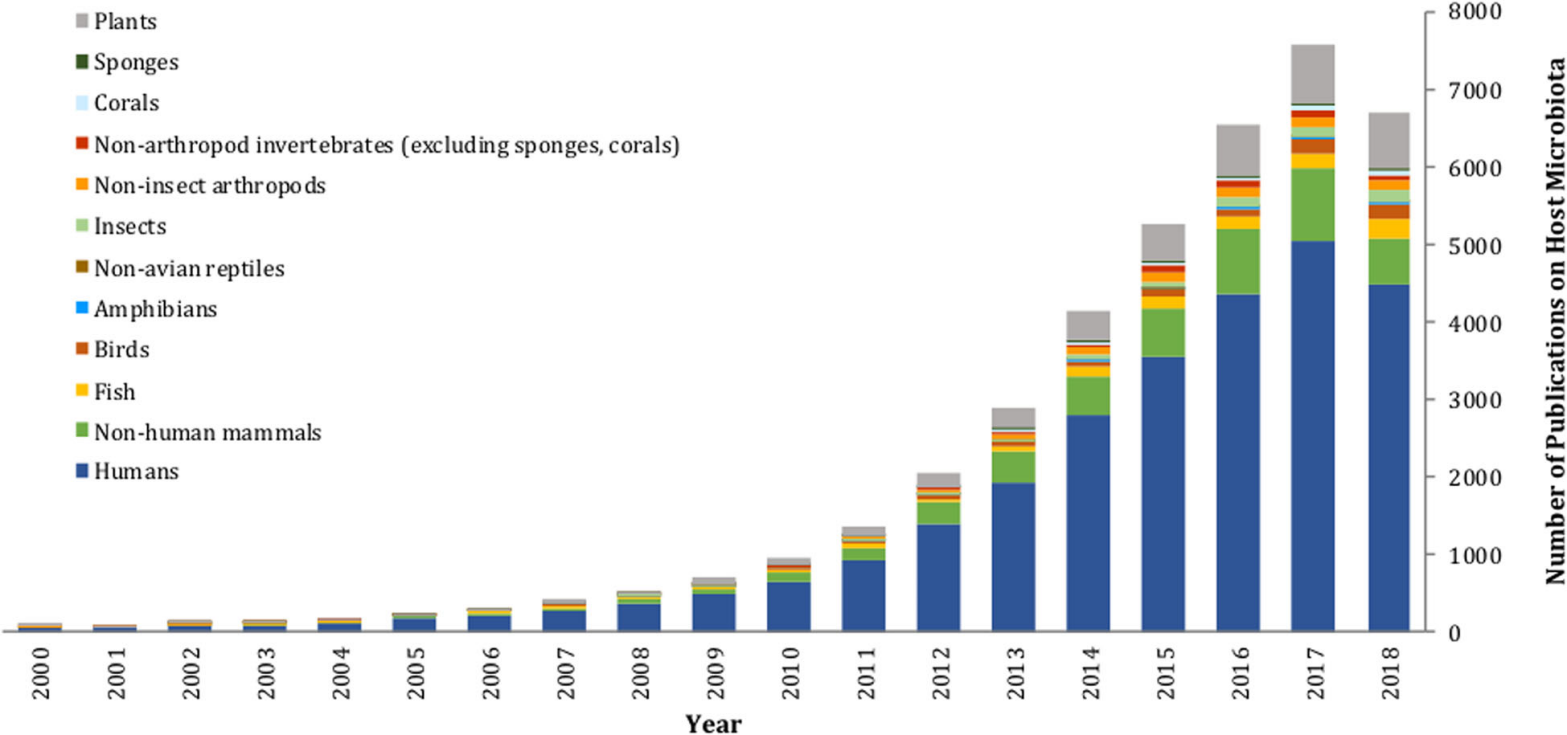
Trends in published host-microbiome studies through time. Data based on a custom keyword statements within NCBI PubMed

**Fig. 3:**
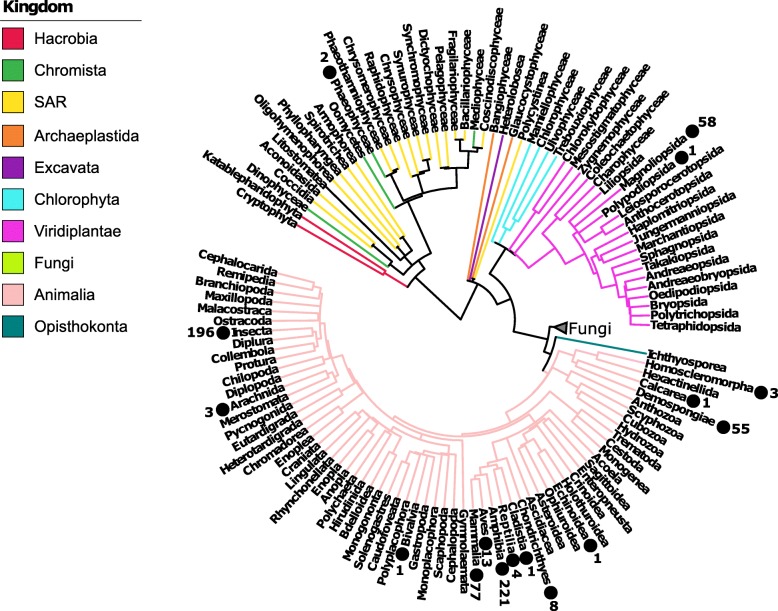
Phylogenetic tree of selected eukaryotic hosts at the class level. Numbers adjacent to black circles indicate the number of species included in our dataset from that class. Groups’ missing microbiome data are evident; however, only studies focusing on the V4 region of the rRNA gene were included. Tree was retrieved from TimeTree (http://www.timetree.org), which aggregates taxonomic and phylogenetic information from published literature. Interact with this tree at IToL: https://itol.embl.de/tree/1306494203341921544122745

Meta-analysis of microbiomes through the growing body of next-generation sequencing data represents a new tool for ecologists and is a systematic approach to combining the results of multiple studies and synthesizing relevant data to gain new insights [4, 24]. This technique allows the synthesis of regional- and local-scale data to elucidate global-scale patterns of microbial community structure, function, and interaction, with indications for public health and extending to environmental policy [25]. Meta-analysis has been increasingly recognized as an important scientific approach, with many prominent researchers proposing standards for, and encouraging, its continued widespread use [25–27]. Human microbiomes have been the target of several meta-analyses, revealing insights that indicate microbial involvement in health as well as disease [28], and determining core microbiota associated with body sites [29]. Some meta-analyses have synthesized data in order to investigate disease, physiologic, and developmental states with large effect sizes [30, 31]. Meta-analyses of non-human host taxa have found a potential link of convergent microbial symbioses between fish and mammals with salinity and trophic level being important drivers of fish gut microbiomes [32]. While diet also impacts mammal gut microbiomes [6], a study of 18 wild non-human primates indicated that the influence of host physiology and phylogeny was much stronger than diet [33]. Host selection also was found to be more important than diet or captivity status in avian guts [34]. Some key questions for microbiome meta-analyses are featured in Table 1, and while host-microbiome datasets are accumulating with time (Fig. 2), knowledge gaps are identified including gaps in regions sampled (Fig. 1b) and host groups with unstudied microbiomes (Fig. 3). Importantly, the eukaryotic and viral components of the microbiome remain a research frontier. The bacterial microbiome dataset and metadata accumulated here is a public resource and may provide future eco-evolutionary, veterinary, or medical insights. As an example, we investigate the bioclimatic correlates of the abundance of an arthropod symbiont that is increasingly important for disease vector control (*Wolbachia* [59, 60]) across the global dataset we assembled.

**Table 1:** Outstanding questions in host-microbiome research. Host-microbiome research is an emerging field. Knowledge gaps include the eukaryotic and viral components of the microbiome [35–37], novel bacterial clades and uncultured microbes [38–40], and large gaps in the geography and host taxa sampled for microbiome studies. Most studies to date have focused on human or other mammalian gut microbiomes, agricultural plants, and fish studies focused on aquaculture, leaving other vertebrate and invertebrate hosts underrepresented. Wild samples are needed to overcome alterations due to captivity [41, 42]. Recent efforts to place microbiomes within a macroecology context described patterns across scales [43], or metacommunity or community ecology contexts to learn about microbial migration [44, 45], community assembly and succession [46], and functions for host health [12, 47–49]

Frontiers in host-microbiome research	
1) Are there dormant and active microbiome subsets of the host microbiome, and how do these subsets change with environmental conditions [50]? How does microbial antagonism and interaction protect hosts or facilitate host invasion [11]?	
2) What are the effects of environmental change on colonization, dysbiosis, or adaptive microbiomes? Do abiotic conditions have stronger effects on ectotherms compared with endothermic hosts? Are microbial therapies effective [17, 51–53]?	
3) What is the significance of core (stable through time and prevalent among individuals) vs peripheral (transitory or rare) microbiomes or gene functions including metabolic pathways in host populations, and is there a trade-off or shift in core microbiome with host immunity, anatomy, life stage, or environmental conditions? Are core microbiomes likely to be of use in personalized medicine or disease diagnostics? Are core microbiomes, particularly of non-human hosts, lost with industrialization [31, 54, 55]?	
4) Metabolomics and functional analyses are a research frontier; do they require a renewed focus on culture-based research and genome sequencing [56–58]?

## Results

After combining microbiome datasets targeting the V4 region of 16S rRNA gene obtained from Illumina platforms and using a standard analysis pipeline (Fig. 1a) [61] to identify sub-Operational Taxonomic Units (sOTUS), or unique sequence variants [27], we were able to compare data from 654 host species distributed globally. Samples ranged from 46 countries in 7 biomes and from hosts including marine sponges 490 m below the sea surface to bar-headed geese at 3955 m elevation in the Himalayas. While representing a global sampling of hosts, visual inspection of the map of global distribution of samples suggests that hosts from much of Africa and Asia are underrepresented (Fig. 1b), and many eukaryotic clades have not been sampled, thus providing ample opportunities for future research (Fig. 3). We subset the 15,790 samples into 3 non-overlapping groups for separate analyses: internal (*N* = 741 samples), external (*N* = 1193), and marine external microbiomes (*N* = 266; Table 2, Fig. 1a). Given what has already been described by the Earth Microbiome Project, separate analyses are warranted based on host ecosystem type (marine as distinct from terrestrial/aquatic) and host internal or external body site [3].

**Table 2:** Summary statistics and metadata fields for the full dataset, partitioned for analyses by internal or external microbiomes of terrestrial and freshwater host organisms

Metadata field	Description	Full dataset	Internal	External	Marine_external
Samples	sample runs included in this study	15,790	741	1193	266
sOTU	exact sequence variants (sub-operational taxonomic units)	175,709	17,544^a^	28,410^a^	5.077^a^
Publications (by doi)	digital object identifier accession number of published studies	51	23	13	6
Host Kingdom	https://www.itis.gov or http://www.eol.org	2	2	2	2
Host Phyllum	https://www.itis.gov or http://www.eol.org	8	3	2	4
Host Class	https://www.itis.gov or http://www.eol.org	16	7	5	8
Host Order	https://www.itis.gov or http://www.eol.org	80	26	23	27
Host Family	https://www.itis.gov or http://www.eol.org	177	65	52	45
Host Genus	https://www.itis.gov or http://www.eol.org	427	171	106	58
Host species name	full scientific name of host organism	654	204	239	78
Host taxid	taxa id number for the host species from NCBI Taxonomy Browser	640	197	236	78
Collection timestamp	date of sampling in DD/MM/YYYY format	1069	143	173	108
Countries	country from which samples were collected	46	26	18	20
Latitude range (deg)	latitude in decimal degrees	-43.53 to 60.17	-37.94 to 60.17	-39.84 to 52.28	-43.14 to 51.73
Longitude range (deg)	longitude in decimal degrees	-157.79 to 174.83	-121.79 to 152.31	-122.83 to 138.94	-157.79 to 174.83
Elevation range (m)	GPS coordinates used to estimate elevation if not stated in study	-490 to 3955	-3 to 3955	17 to 3837	-490 to 25
microbial_habitat_type	internal, external, and whole organism	3	internal and whole organism	external	external
internal_habitat_type	digestive-associated, oral, nasal, lung, reproductive, leaf internal, root internal, and n/a	8	1	n/a	n/a
digestive_habitat_type	foregut, fecal, cloacal, intestine, stomach, other and n/a	7	5	n/a	n/a
external_habitat_type	leaf surface, root surface, animal surface, gill, and n/a	5	n/a	4	3
surrounding_habitat	freshwater, marine, terrestrial	3	freshwater,terrestrial	freshwater,terrestrial	marine
lifestage	adult, juvenile/pupae, larvae, and infant	4	4	3	2
sampling_month	month when the samples were collected	12	12	12	12
trophic_diet	scaled 0=primary producers, 1=herbivore, 2=omnivore, 3=carnivore, 4=detritivore/scavenger	5	5	n/a	n/a
preservation_method	sample storage by direct freeze, ethanol, RNA-later, or other	4	4	3	2
extraction_method	name of DNA extraction kit	15	5	6	2
biogeo_realm	https://en.wikipedia.org/wiki/Biogeographic_realm	7	6	5	4
Worldclim2 bioclimatic variables	http://worldclim.org/version2; Fick et al 2017	n/a	8	8	n/a
Marine geophysical variables	http://marspec.weebly.com/about.html; Sbrocco et al 2013	n/a	n/a	n/a	11
Immune Complexity (binary)	inferred from host class information	n/a	2	2	n/a
Immune Complexity (ordinal)	scale based on Flajnik et al 2018;	n/a	1-9	1-9	n/a

^a^sOTUs after rarefaction at 1000 reads per sample

For marine organisms, our analysis was limited to available external samples. For terrestrial/aquatic organisms, we divided analyses between internal and external samples. Internal microbiomes were primarily associated with the digestive system or whole organism samples in the case of insects. While the microbial communities in insects can differ with surface or organ sampled, for the sake of our analysis, we classified whole organism tissues as internal (Fig. 1a). This is consistent with descriptions of gut microbiomes driving the community structure of whole-organism samples [62], and the lower quantity of bacterial cells on human skin vs. large intestine, for example [63]. External microbiomes were sampled from host surfaces including the skin, gill, or leaves, and analyzed separately.

**Fig. 4:**
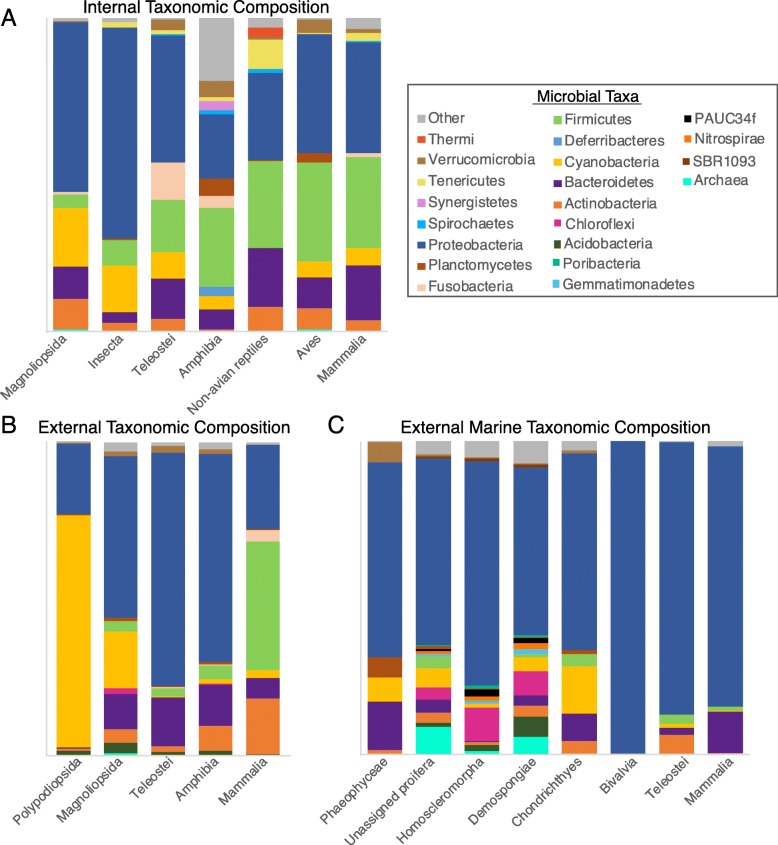
Taxonomic and function composition of host microbial communities across host classes and microbial habitats. **a** Internal microbiomes of terrestrial and freshwater organisms, **b** external microbiomes of terrestrial and freshwater organisms, and **c** external microbiomes of marine organisms. Each color represents a unique bacterial phylum. A legend for microbial taxa including bacterial phyla and Archaea is provided

We assembled an extensive collection of metadata for each sampled host, including host taxonomy, life stage, trophic level, and body site sampled, as well as environmental factors for each sampling location such as macroclimate metrics of temperature and precipitation, land cover, and elevation. We then visualized the dominant microbial taxa from each host class separately by host habitat and body region (internal, external; Fig. 4, Additional file 1: Figure S3), and performed analyses of alpha and beta diversity metrics. Results of all analyses can be reproduced with the data included in Additional files 2, 3, 4, 5, 6, 7, 8, 9, 10, 11, and 12 (see the “Methods” section and Additional file 1 for a description of the data files provided), or re-analyzed in the future as bioinformatics techniques continue to improve.

**Fig. 5:**
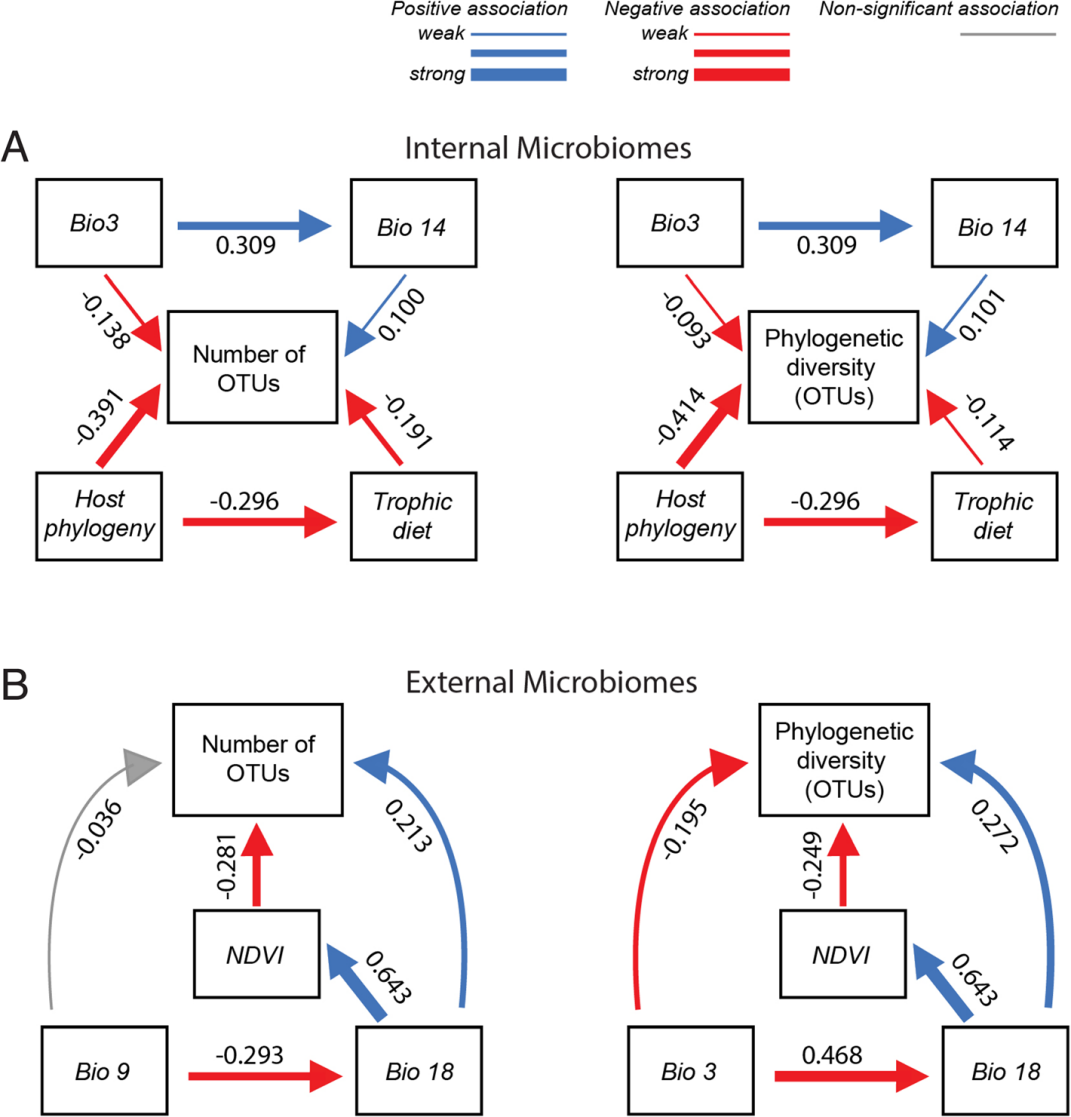
Path analyses showing direct and indirect effects of the best abiotic and biotic predictors of number of sOTUs (left) and phylogenetic diversity (right). Models explaining internal (**a**), and external microbiome diversity (**b**) are shown. Numbers are standardized path coefficients (*P < 0.05). Blue arrows depict positive associations whereas red arrows depict negative effects. Gray arrows depict non-significant paths. The thickness of the arrows represents the relative strength of each relationship. Bioclimatic variables include the following: Isothermality (Bio3), Mean Temperature of Driest Quarter (Bio9), Precipitation of Driest Month (Bio14), and Precipitation of Warmest Quarter (Bio18)

To analyze alpha diversity, including richness (number of sOTUs) and phylogenetic diversity of sOTUs, we carried out model selection (Additional file 1: Table S1), resulting in a reduced set of variables for inclusion in downstream path analyses: a temperature metric and a precipitation metric, normalized difference vegetation index (NDVI), and host phylogeny and trophic diet. Path analysis was used to test for the magnitude and significance of hypothesized causal connections and to determine direct vs. indirect influences on alpha diversity. Separate path models were run to examine how variables interacted to impact microbiome phylogenetic diversity or richness and were performed separately for internal and external microbiomes (Fig. 5). Latitude was excluded from path analyses because of multicollinearity with climatic variables (analyzed separately in Additional file 1: Figure S2).

**Fig. 6:**
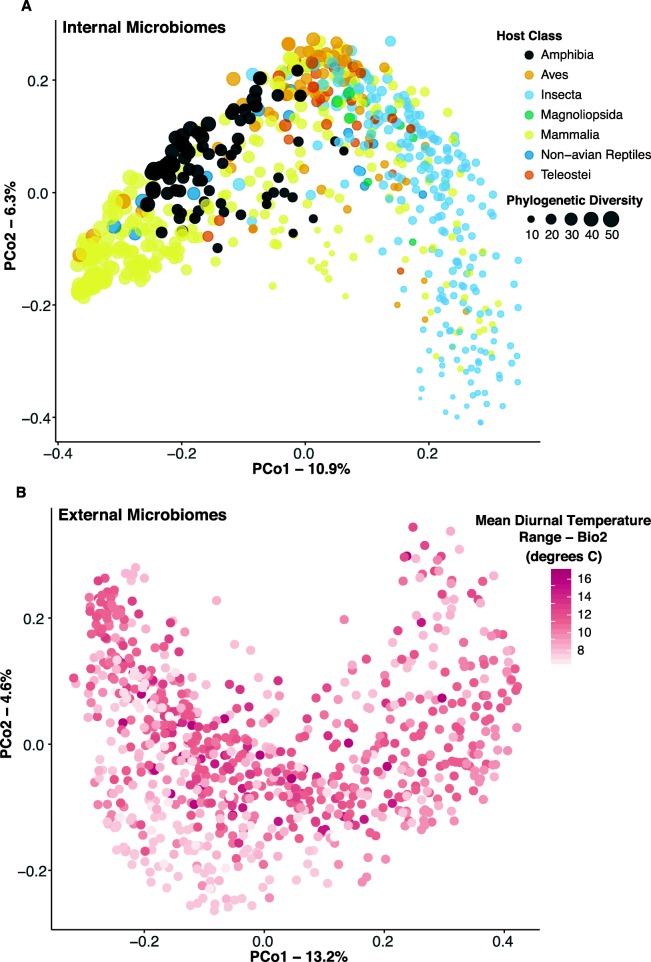
Principal coordinates analysis of Unifrac distances. **a** Internal microbiomes, colored by host class, and size-scaled by microbial phylogenetic diversity. Host class explained 13.9% of the variation in community structure (Additional file 1: Table S2). **b** External microbiomes, color scale white-red corresponding to low-high Mean Diurnal Temperature Range (Bio2; Mean of monthly (max temp–min temp)). Bio2 explained 59.6% of the variation in external microbiome community structure (Additional file 1: Table S2)

Patterns of beta-diversity were analyzed by permutational analysis of variance (PERMANOVA) to determine variables of greatest importance in structuring the microbial communities (Additional file 1: Table S2). Internal microbiomes were most significantly structured by host class (explaining 14% of the variation), as well as trophic diet, several bioclimatic factors, latitude, elevation, and NDVI (Additional file 1: Table S2). A principal coordinates analysis illustrates clustering of internal microbiome by host class and depicts the higher microbial diversity found in samples of mammals, amphibians, birds, and non-avian reptiles (crocodile and iguana) as compared to insects and carnivorous plants (Fig. 6a; Additional file 1: Tables S2, S3). External microbiomes were structured most strongly by bioclimatic factors (bioclim2 and bioclim15 explaining 60% and 7% of variation, respectively), as well as external habitat type (5%) as compared to host class (not significant; Additional file 1: Table S2). Bioclim2 is a measure of mean diurnal temperature range and most significantly structured the external microbiome (Fig. 6b, Additional file 1: Figure S3). Because our dataset was heavily filtered to standardize sampling among host species and locales, we verified that we retained power to detect previously described patterns. For example, four human populations included in the dataset recapitulated previously described patterns including greater gut microbiome diversity in developing countries ([64]; Additional file 1: Figure S4).

**Fig. 7:**
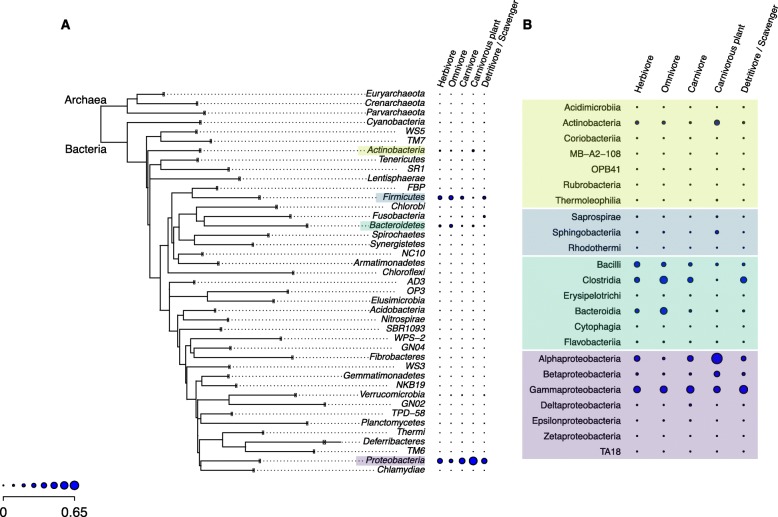
Bacterial abundance across trophic diets. **a** Phylogenetic tree of major bacterial phyla and their abundance by trophic diet for internal microbiota. The size of the circle depicts the proportion of a given bacterial group within the community by the trophic diet. **b** Abundance of major bacterial classes of selected bacterial phyla across trophic diet for internal microbiota

Because host class had the strongest association with the internal microbiome structure, we examined this relationship in greater detail, hypothesizing that predicted function may provide further discrimination. We used PICRUSt (Phylogenetic Investigation of Communities by Reconstruction of Unobserved States) to examine functional properties of the internal microbiome [21]. We characterized the accuracy of this tool by comparing the weighted Nearest Sequenced Taxon Index (weighted NSTI) score across host classes and filtering samples above the cutoff score of 0.06 (Additional file 1: Figure S5). The major functional categories across host taxa included membrane transport, amino acid metabolism, and carbohydrate metabolism (Additional file 1: Figure S6). Functional capacities of the internal microbiomes were structured by similar variables as the microbial communities including host class (explaining 13% of variation) as well as trophic diet, bioclimate variables, latitude, elevation, and NDVI (each explaining less than 5% of variation; Additional file 1: Table S2). Trophic diet was a significant factor of ecological interest, though explaining little variation. Some host taxa including Amphibia and Teleostei demonstrate developmental shifts in both trophic level and microbiome [46, 65–67]. A phylogenetic tree of internal microbiome phyla illustrated potential trends by host trophic level/diet in abundance of major groups such as decreasing *Proteobacteria* and increasing *Firmicutes* at higher trophic levels (Fig. 7). Profiles of carnivorous plants were distinct from animal carnivores. Shifts in major classes with four bacterial phyla are illustrated in Fig. 7b.

**Fig. 8:**
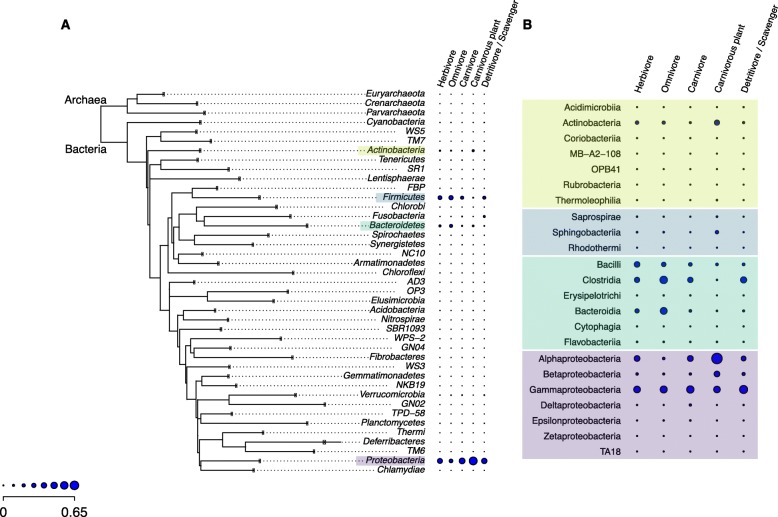
Immune system complexity associations with diversity of host microbiomes. **a** Mean richness and phylogenetic diversity (external and internal microbiomes) for host genera with adaptive immune systems is significantly greater than host genera with only innate immunity. *P < 0.001, Wilcoxon tests. **b** Mean internal sOTU richness correlates with adaptive immune system complexity based on the Flajnik [68] comparative immunology scale (see Additional file 1: Table S3)

**Fig. 9:**
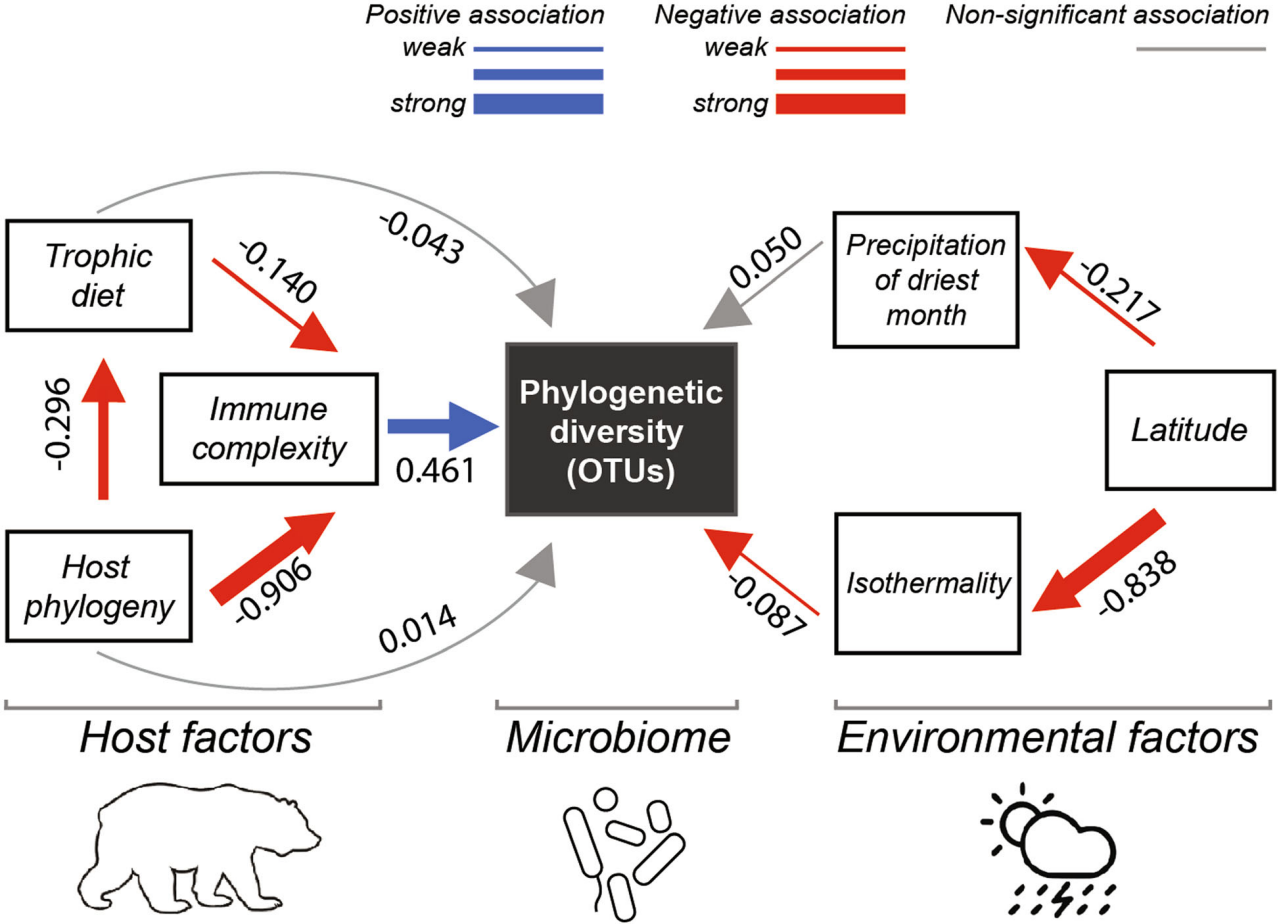
Path model of internal microbiomes depicting direct and indirect effects of immune complexity in the context of the best biotic and abiotic predictors of microbial phylogenetic diversity. Numbers are standardized path coefficients. Blue arrows depict positive associations whereas red arrows depict negative effects at *P* < 0.05. Gray arrows depict non-significant paths. The thickness of the arrows represents the relative strength of each relationship

Eco-evolutionary patterns in immune complexity were examined to test a mechanistic hypothesis explaining trends in host microbiome diversity. Hosts with adaptive immune systems had significantly greater microbiome richness and phylogenetic diversity than hosts with only innate immunity (Wilcoxon, *P* < 0.001; Fig. 8a). Complexity of host immune systems at a broad level was correlated with microbiome diversity (Fig. 8b). While strongly correlated with host phylogeny, the complexity of adaptive immune systems across hosts based on a review by Flajnik [68] was developed into a matrix and score for each host class (Additional file 1: Table S3). Inclusion of the scale of adaptive immune system complexity in the path model for internal microbiomes indicated a significant direct association with microbial phylogenetic diversity (Fig. 9).

The role and maintenance of core members of host microbiomes remains a research frontier, and we hypothesize a trade-off between immune complexity and abundance of core microbes defined specifically at the strain level (Table 1). A preliminary analysis based on a permissive definition of core bacteria (80% prevalence among samples) is presented in Additional file 1: Table S3.

**Fig. 10:**
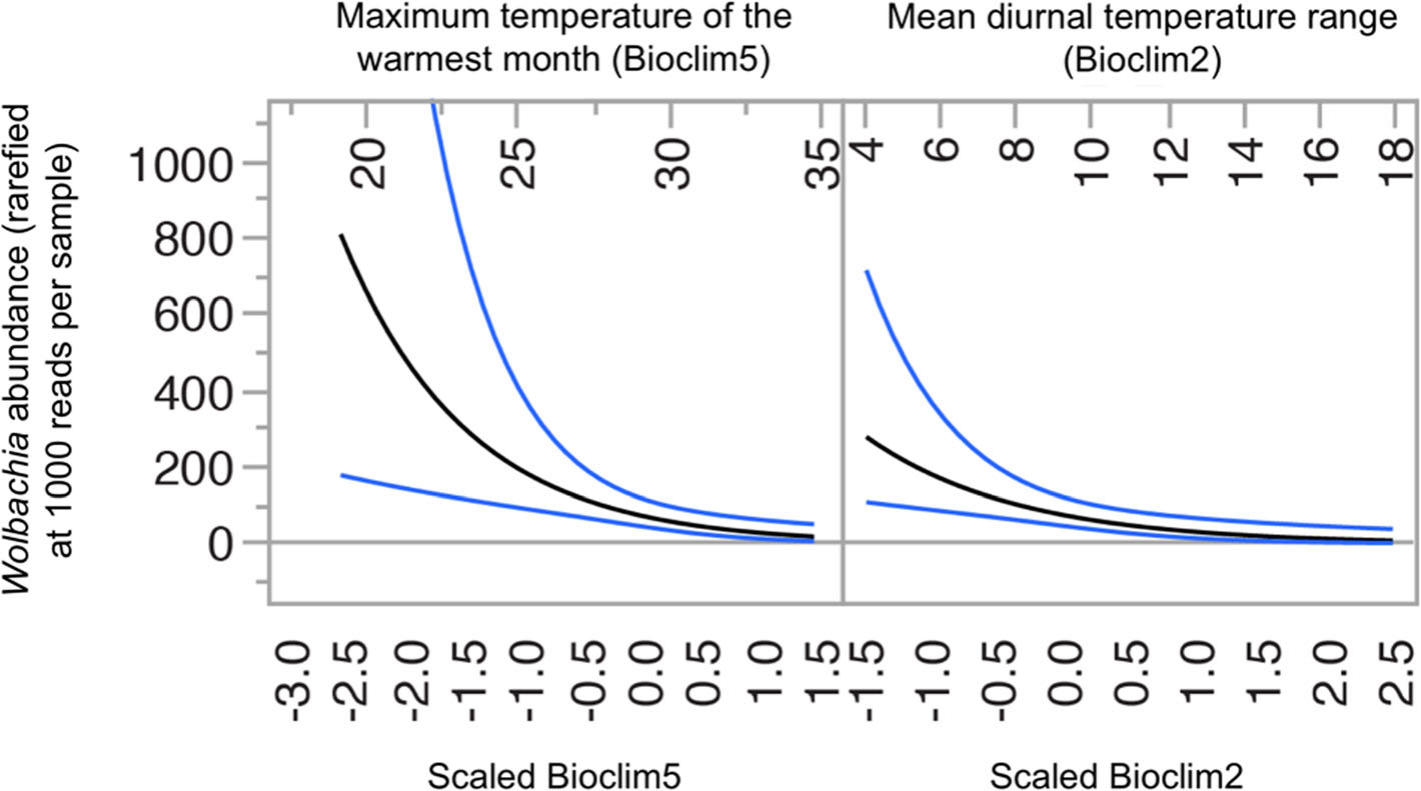
*Wolbachia* in insects are globally diverse and decrease in abundance with temperature range. Maximum temperature of the warmest month (Bioclim5) and mean diurnal temperature range (Bioclim2) negatively predict relative *Wolbachia* abundance in samples derived from insects. Blue lines indicate 95% confidence limits. Details can be found in Additional file 1: Figure S8. This is one example of how this global microbiome dataset can be used to better understand and analyze host-microbe interactions

The dataset we assembled provides a broad overview of factors driving host microbiome structure, function, and diversity (Figs. 4, 5, and 6). It also provides a resource for comparative microbial ecology. As an example of its utility, we scrutinized the dataset for global trends in the distribution of *Wolbachia*, a genus of common Gram-negative bacteria known to be reproductive parasites and serving as mosquito disease vector control agents; our data confirm these bacteria being mainly present in insects (Table 3) and find them most abundant in cool environments of the globe (Fig. 10). At a global scale, we thus describe novel patterns and lay the groundwork for future mechanistic studies on host-microbiome interactions.

**Table 3:** Taxonomic classes with positive detection of *Wolbachia*-specific sOTUs

Taxonomic class	Average reads per sample^a^	Number of unique *Wolbachia* sOTUs within class (total = 33)^b^	Percentage of *Wolbachia* sOTUs found in Insecta^c^
Amphibia	0.131	12	41.7
Demospongiae	0.007	1	100.0
Insecta	33.305	23	100.0
Magnoliopsida	0.024	1	0.0
Mammalia	0.002	4	75.0
Phaeophyceae	0.003	1	100.0

^a^Insecta had a substantially higher number of average *Wolbachia* reads than any other class. Most other *Wolbachia-*positive samples were rare and found in organisms where insects are a substantial portion of the diet (e.g., amphibia, bats, carnivorous plants), and as some of the host samples were derived from the gut, this is to be expected. Interestingly, Demospongiae were positive for *Wolbachia*, which may indicate that there were marine arthropods living within the sponges that were *Wolbachia*-positive

^b^Insecta showed the highest number of unique *Wolbachia* sOTUs present within each class. Amphibia was high in the number of unique sOTUs, and many of these amphibian samples were taken from the gut

^c^Calculating the relative percentage of sOTUs found within each class that are also found within class Insecta indicated that the majority of sOTUs attributed to other host samples can be found in Insecta (likely as part of the diet of the host), and the remaining sOTUs are likely insects that are not present as directly collected samples in the dataset that were also prey items to their hosts. A heatmap showing mean abundance of each *Wolbachia* sOTU is presented in Additional file 1: Figure S8

## Discussion

The assembly and composition of host-associated microbiomes are attendant with forces of ecology, evolution, host physiology, and immune function. Here, we examine microbiomes from 654 host species and begin where our current understanding trails off. That is, at the broadest scale, microbiomes can be differentiated first by free-living or host-association according to the Earth Microbiome Project Ontology ([4]; Additional file 1: Figure S1), likely driven by both biotic and abiotic pressures. Salinity has a large effect on free-living microbiomes [4], and here, we analyze microbiomes on external host surfaces of marine organisms separately from those of terrestrial and aquatic hosts. Many other factors influence or interact with host microbiomes, and here, we examine whether these factors differ depending on whether the microbiome is from external host surfaces such as leaves, gill, or skin, or is internal to the host, including communities in the digestive and reproductive systems. Host immune complexity, though strongly correlated with host phylogeny, appears to influence both internal and external microbiomes in terms of diversity and community structure (Figs. 8 and 9). Indeed, internal microbiome phylogenetic diversity had a weak direct path from host phylogeny, but a strong indirect path from host phylogeny through immune complexity (Fig. 9).

Our analysis revealed that internal (digestive-associated) microbiome diversity was predominantly shaped by host factors, while external (surface-associated) microbiome diversity was strongly associated with normalized difference vegetation index (NDVI) and some climatic factors such as precipitation of the warmest quarter (Bioclim18; Fig. 5). Similarly, a recent study of amphibian skin microbiomes found higher diversity on hosts in environments with colder winters and less stable thermal conditions (i.e., temperate regions [5];). Thus, increasing climatic variability appears to promote coexistence and diversity of external microbiomes, perhaps through ecological succession or dormancy mechanisms [5]. Nottingham et al. [69] found that both plant diversity and soil microbiome diversity follow temperature (and elevation) gradients at a regional scale with more species under warmer conditions. At a global scale, the external plant microbiome richness was also negatively correlated with elevation (Pearson’s correlation, *N* = 85, *r* = − 0.324, *P* = 0.002), but mean annual temperature was not significantly correlated with these plant microbiomes (*r* = − 0.174, *P* = 0.111). Path analysis of our external microbiome dataset containing 33 plant host species did not show a significant effect of temperature on microbiome richness, but rather greater diversity with lower NDVI (Fig. 5). While host factors did not play a significant role in external microbiomes at the global scale, they can be important divers of host microbiomes locally (e.g., [70]).

Internal microbiome diversity was most strongly driven by host factors including host phylogeny and, to a weaker extent, diet or trophic level (Figs. 4, 5, and 6). Similarly, a study focused on non-human primates found that host phylogeny had a larger impact than dietary niche on gut microbiomes [33]. This finding is also supported by studies in other taxa including larval amphibians and aquatic invertebrates [71]. Previous studies found a more prominent role of host diet in shaping the gut microbiome [72, 73], particularly within clades such as lizards [74], fish [32, 67, 75], birds [76, 77], or mammals [78]. Captivity may produce bottom-up effects on microbiomes by altering environmental conditions and diets from field conditions [41]. Our data mirror macroecological patterns (e.g., Paine’s keystone predation [79];) and suggest that bottom-up processes of nutrient conditions shaped by host diet have weaker effects on microbiome diversity and structure than do the top-down processes of host physiology and immune function. Just as abiotic factors can influence or reverse top-down effects of predators on ecosystem function [80], the relative strength of host immune function may be similarly disrupted (e.g., antibiotics, immunocompromise) and have strong influences on the host microbiome ([15, 17, 31]; Table 1).

Host immune systems are thought to function in reducing microbes, commonly maligned as germs. We found, perhaps counterintuitively, that a greater diversity of bacteria are hosted by organisms with more complex adaptive immune systems (Figs. 8 and 9). Indeed, we would add to Mcfall-Ngai’s [1] prescient framework quoted above and suggest that core bacteria that are more abundant in organisms with exclusively innate immunity may trade off this stability with more complex immune function during the evolution of adaptive immunity [68]. Indeed, top-down effects of immune function may be analogous to predation effects and promote maintenance of diversity by decreasing competition and allowing coexistence [79]. Understanding these patterns may encourage forward thinking responses to current environmental impacts affecting microbiome evolution and host health ([81, 82]; Table 1).

## Conclusions

Several reviews have highlighted the need for standardized data collection methods and greater taxonomic breadth and sampling of wild hosts, considered the least investigated compared with domestic and model host species [67, 81, 83]. Wild hosts may provide the greatest insight on host evolutionary biology and ecology, since captivity can have dramatic impacts on the microbiome [41, 42]. Understanding how host-associated microbiomes are naturally assembled and influenced by abiotic and host conditions provides insight on potential reservoirs of microbiota and can inform metacommunity models that predict community structure and transmission of microbes or dispersal and feedback between hosts and habitats [44, 84]. While there remain wide geographical (Fig. 1) and taxonomic gaps (Fig. 3) in host-microbiome studies, our large sample set enabled the description of global-scale patterns in both internal (gut and reproductive tract) as well as external surface (leaves, skin, gill) microbiomes. Using a standardized approach, we found compelling evidence that internal vs. external microbiomes differ in the predominant factors associated with diversity and composition. Our analysis also revealed several correlations that at first glance were counterintuitive, such as microbiome diversity increasing with host immune system complexity, that suggest previously unrecognized top-down regulating effects. The analysis of *Wolbachia* occurrence and diversity across hosts and environments exemplifies the data mining potential of the metadata set assembled for this study. We anticipate that the identified patterns will be instrumental in deriving testable hypotheses and therefore have great potential to stimulate exciting experimental tests to elucidate the underlying mechanisms.

## Methods

### Sequence acquisition

To investigate global patterns of microbiome diversity and structure across host systems, we performed standardized bioinformatics analysis on combined datasets and procured environmental and host-associated metadata. We focused on studies with targeted 16S rRNA gene sequence data obtained from an Illumina platform. A literature search was conducted to identify relevant studies with data accessible from public databases. The majority of sample sequences were downloaded from the Sequence Read Archive (SRA) of the National Center for Biotechnology Information (NCBI), the European Nucleotide Archive (ENA), MG-RAST, and Qiita repositories. The combined dataset is made available here (Additional file 2, 3, 4, 5, 6, 7, 8, 9, 10, 11, and 12), and accession numbers and DOIs for all published studies are indicated in the mapping (metadata) files described in Additional file 1.

Datasets were selected that followed the Earth Microbiome Project (EMP) protocols and standards (http://www.earthmicrobiome.org/protocols-and-standards/). Studies were first screened to ensure certain criteria were met, including (1) collected from a “wild setting,” where hosts were not exposed to any experimental treatments; (2) ethanol preserved or frozen until DNA extraction; (3) targeted the V4 region of the 16S rRNA bacterial gene region, using primers 515f and 806r [85]; and (4) sequencing on Illumina MiSeq or Illumina HiSeq platforms. Once a study passed our inclusion filters, we downloaded fastq files and obtained metadata for each study. The compiled dataset is comprised of 15,790 samples from 51 studies including all host-associated data from the Earth Microbiome Project [4] and includes 16 host classes (Fig. 3) from plants to corals to vertebrates (including 4 human populations; Additional file 1: Figure S4, [9, 50–58, 85–121]).

### Sequence preparation

Sequence data were quality filtered using Quantitative Insights into Microbial Ecology (QIIME) defaults and classified into sub-Operational Taxonomic Units (sOTUS, or unique sequence variants) using the Deblur workflow [27]. Within Deblur, reads were trimmed to 90 bp to correspond with the shortest read length in the combined dataset. Taxonomy was assigned using the RDP Classifier with GreenGenes 13-8 as reference through a custom bash script [122, 123]. Then, sOTUs with fewer than 90 reads, taxonomically assigned as “mitochondria” and “chloroplast,” or not identifiable at the Kingdom level were removed. A phylogenetic tree was built with fasttree2 [124]. Samples were rarefied at 1000 sequences per sample to retain most samples, normalize read counts across samples, and reduce computational demands. Furthermore, we standardized sampling across locations and host species by randomly selecting up to 10 samples from a given host species at a given country (see Table 2 for overall sample sizes and number of unique sOTUs). Datasets before and after processing as described above can be found in the Additional files.

### General analysis framework and datasets

We employed multiple statistical approaches, including general linearized models, permutational multivariate analysis of variance, and path models, to explore the main drivers of host-associated microbial diversity, community structure (i.e., beta diversity), and PICRUSt-predicted functionality. Tests also targeted hypotheses surrounding how immune function may shape diversity and composition of host microbiomes and how the distribution of *Wolbachia* varies across bioclimates.

For our analyses, the data were divided into 3 main subsets: (i) an *internal* microbiome dataset comprised of 741 samples derived from internal host habitats or whole-body samples, (ii) an *external* microbiome dataset comprised of 1193 samples derived from external host surfaces, and (iii) a *marine* dataset comprised of 266 external surface samples from marine organisms. Table 2 provides detailed information on sample types included in each dataset. The geographic distribution of these samples is depicted in Fig. 1. Additional data files for each subset are provided as indicated in Additional file 1.

### Sample metadata and predictor variables

For each sample, we tabulated a comprehensive set of abiotic and biotic predictor variables for testing targeted hypotheses and inclusion in model-based analyses of our datasets. Associated metadata included in the mapping file was gathered by downloading the metadata for each study, taken directly from the paper corresponding to the study, or obtained from publicly available databases. Table 2 provides a description of each metadata field.

Elevation data was extracted from Google Earth using latitude/longitude coordinates. Current bioclimatic variables representing temperature and precipitation (1 km resolution) were extracted for each unique geographic location from the WorldClim2 database. These 19 bioclimatic variables were extrapolated from a global network of stations collecting data from 1970 to 2000 [125]. Eighteen marine and geophysical variables were extracted from the MARSPEC database at the same 1 km resolution [126] for the marine dataset models. The variables of interest are defined in Additional file 1: Table S1 and below. Because of expected high correlation between many Worldclim bioclimatic variables, we filtered these predictors to a least correlated subset. From the 19 bioclim variables, we selected the 8 least correlated ones (caret package in R [127]) based on a threshold of *r* < 0.7. These least correlated variables included Mean Diurnal Temperature Range (Bio2), Isothermality (Bio3), Max Temperature of Warmest Month (Bio5), Mean Temperature of Driest Quarter (Bio9), Precipitation of Driest Month (Bio14), Precipitation Seasonality (Bio15), Precipitation of Warmest Quarter (Bio18), and Precipitation of Coldest Quarter (Bio19) and were included in model selection procedures described below. The same procedure was performed for the 18 marine biophysical predictors resulting in the following least correlated subset: bathymetry, east/west aspect (biogeo1), north/south aspect (biogeo2), plane curviture (biogeo3), distance to shore (biogeo5), bathymetric slope (biogeo6), concavity (biogeo7), sea surface salinity of the saltiest month (biogeo10), annual variance of sea surface salinity (biogeo12), sea surface temperature of warmest month (biogeo15), and annual range of sea surface temperature (biogeo16).

Biotic predictors included host identity (host class or host phylogeny), host trophic diet (for internal dataset), external surface type, and immune system complexity. Either host class or a numeric nMDS proxy of host phylogeny was used to represent host identity. Host class information was obtained from the Encyclopedia of Life database (https://eol.org/). The host phylogeny proxy was created via the following steps. First, using timetree.org, we recovered a time-calibrated phylogenetic tree of host species represented in our study [128]. Second, patristic pairwise distances, i.e., branch lengths separating taxa, were then calculated with the Ape and Adephylo packages in R [129, 130]. Third, we performed non-metric multidimensional scaling (nMDS), constrained to one dimension, on the patristic distance matrix in SPSS v24 (IBM Corp, Armonk, NY, USA) to create a univariate host phylogeny variable for use in model-based analyses. The nMDS proxy phylogeny variable was chosen because it captured the phylogenetic relationships among hosts and is a numeric variable available for path analyses. Host trophic diet levels was estimated based on the diet category, including primary producers (level 0), herbivores (1), omnivores (2), carnivores (3), and scavengers/detritivores (4). External surface type information was obtained from data available with sequence archives and the published papers. Immune system complexity was estimated in two ways: (i) as a binary categorical variable indicating whether the host possessed an adaptive or innate only immune system, and (ii) as an ordinal scale derived from immune system characteristics from Flajnik [68] (Additional file 1: Table S3).

### Analysis of alpha diversity

Model selection procedures implementing generalized linear models (GLMs) and comprehensive path models were used to analyze alpha diversity for each of our datasets. We performed GLM model selection procedures to identify which of our main biotic and abiotic variables best predicted richness, measured as number of sOTUs and Faith’s Phylogenetic Diversity for each of our three main datasets. More specifically, for internal, external, and marine microbiomes, we screened a total of 25 explanatory variables, including host phylogeny/identity, trophic diet (internal only), external habitat types (marine only), absolute latitude, elevation, normalized index vegetation index (NDVI; external only), 8 least cross-correlated bioclimatic variables (internal and external only), and 11 least cross-correlated ocean biophysical variables (marine only; see above for least correlated sets). Competing models were ranked based on the Akaike Information Criterion (AIC), and we reported the most parsimonious model for each of our three datasets for each response variable. Multicollinearity was evaluated in each of the final models using the variance inflation factor (VIF) calculated with car package [131] in R.

We applied path analyses with maximum Wishart likelihood (500 iterations) to test for the relative strength of direct and indirect associations among abiotic/biotic predictors and microbiome diversity of our internal and external microbiome datasets. Path models were not built for the marine dataset due to low predictive power of included variables in the GLM model selection. We built models including the most parsimonious set of explanatory variables predicting microbiome diversity according to our GLM model selection procedures (see above). Our four path models explaining number of OTUs and Faith’s Phylogenetic Diversity for both internal and external microbiomes included all ecologically meaningful associations among predictor variables. We performed a fifth path model for Faith’s Phylogenetic Diversity of internal microbiomes, including direct and indirect effects of immune system complexity in the analysis. We reported whole model fit for our path models using the root-mean square error of approximation (RMSEA). When goodness of fit threshold for was not met (RMSEA ≤ 0.1), we sequentially removed the weakest paths until minimum RMSEA threshold was met for each model. Analyses were performed using RAMONA platform in SYSTAT 13.2 [132].

### Analysis of beta diversity

We performed permutational multivariate analysis of variance (PERMANOVA) to analyze microbial beta diversity and identify abiotic and biotic parameters influencing internal, external, and marine microbiomes. Weighted and unweighted Unifrac distances were calculated in QIIME, and PERMANOVA models were implemented in R with the adonis2() function in the vegan package [133]. Independent models were run for the internal, external, and marine datasets. Predictor variables included in beta diversity models were hypothesis-driven and included all variables from the best alpha diversity model for the given dataset as well as selected biotic factors. We visualized overarching patterns in microbial beta diversity through principal coordinates analysis using ggplot2 in R.

Additionally, we visualized the microbial abundance of major bacterial phyla across host trophic diets. A phylogenetic tree, OTU table, and taxonomy table generated in QIIME were consolidated in R script using the phyloseq and ape packages [129, 134]. The function “dotTree” from the R package phytools [135] was used to generate the phylogenetic tree at the phylum level. Microbial abundance of each phylum and the most abundant classes was calculated for each trophic diet level.

### Functional analysis with PICRUSt

Phylogenetic Investigation of Communities by Reconstruction of Unobserved States (PICRUSt) predicts metagenomic function using marker gene (such as 16S rRNA) surveys [21]. GreenGenes 13-5 OTU IDs are required for PICRUSt analyses; therefore, we clustered our internal microbiome dataset sOTUs to Greengenes OTU IDs at 97% similarity in QIIME. A custom R script was used to combine these GG IDs with abundance information to create a PICRUSt-formatted OTU table. We then performed the following steps: normalization by copy number, metagenome prediction, and consolidation of predictions into KEGG pathways. Results were filtered to retain data with an NSTI score of 0.06 or lower; 247 internal samples were represented in the final analysis. Nearest Sequenced Taxon Index (NSTI) is a confidence measure for PICRUSt predictions. Beta diversity calculations (Bray-Curtis and Jaccard dissimilarity) on PICRUSt functional predictions were performed in QIIME and analyzed in R using the adonis2 function from vegan. The PICRUSt package is available at http://picrust.github.io/picrust/.

### Analyses on immune system complexity

We assessed the effect of immune system complexity on microbial diversity by (a) comparing sOTU richness and phylogenetic diversity between samples from hosts with adaptive vs. innate only immune systems for both the internal and external dataset—pairwise comparisons were implemented with Wilcoxon tests in R; (b) performing a correlation test between microbial richness and immune complexity using Kendall Tau correlation statistics in R; and (c) running our comprehensive path model including the immune complexity scale variable derived from Flajnik [68].

## Supplementary information

**Additional file 1.** Supplementary tables and figures [136–158].

**Additional file 2.** Full metadata file (15,790 samples), unfiltered.

**Additional file 3.** Filtered metadata file for internal microbiomes (741 samples).

**Additional file 4.** Filtered metadata file for external microbiomes (1193 samples).

**Additional file 5.** Filtered metadata file for marine external microbiomes (266 samples).

**Additional file 6.** Deblur sOTU table (biom file) for the full dataset, not rarefied.

**Additional file 7.** Deblur sOTU table (biom file) for the internal microbiome (741 samples), rarefied to 1000 reads/sample.

**Additional file 8.** Deblur sOTU table (biom file) for the external microbiomes (1193 samples), rarefied to 1000 reads/sample.

**Additional file 9.** Deblur sOTU table (biom file) for the marine external microbiomes (266 samples), rarefied to 1000 reads/sample.

**Additional file 10.** RepSet fasta file for the full dataset.

**Additional file 11.** Fasta file to match OTU number with deblur sequence ID.

**Additional file 12.** Phylogenetic tree file used for analyses on data subsets.

**Additional file 13.** Review history.

## Data Availability

The datasets supporting the conclusions of this article are included within the article and its Additional files (Additional file 2 through Additional file 12). Additional file 1 provides descriptions of the data files (metadata, biom, fasta, and tree files). Links to DOI numbers and data repositories for each of the 51 datasets are indicated in Additional file 2. The data were described in the Earth Microbiome Project [4], the Global Amphibian Project [5], and additional recent studies referenced above.
