# Publisher Correction: Host-associated microbiomes are predicted by immune system complexity and climate

**DOI:** 10.1186/s13059-020-01955-y

**Published:** 2020-02-20

**Authors:** Douglas C. Woodhams, Molly C. Bletz, C. Guilherme Becker, Hayden A. Bender, Daniel Buitrago-Rosas, Hannah Diebboll, Roger Huynh, Patrick J. Kearns, Jordan Kueneman, Emmi Kurosawa, Brandon C. LaBumbard, Casandra Lyons, Kerry McNally, Klaus Schliep, Nachiket Shankar, Amanda G. Tokash-Peters, Miguel Vences, Ross Whetstone

**Affiliations:** 1 Department of Biology, University of Massachusetts Boston, Boston, MA 02125 USA; 2 Smithsonian Tropical Research Institute, Roosevelt Ave. Tupper Building – 401, 0843-03092 Panamá, Panama; 3 Department of Biological Sciences, The University of Alabama, Tuscaloosa, AL 35487 USA; 4 School for the Environment, University of Massachusetts, Boston, MA 02125 USA; 5 Animal Health Department, New England Aquarium, Boston, MA 02110 USA; 6 Center of Excellence in Biodiversity and Natural Resource Management, University of Rwanda, RN1, Butare, Rwanda; 7 Zoological Institute, Braunschweig University of Technology, Mendelssohnstr. 4, 38106 Braunschweig, Germany

**Publisher Correction to: Genome Biol (2020) 21:23**

**https://doi.org/10.1186/s13059-019-1908-8**

Following publication of the original paper [1], it was reported that an error in the processing of Fig. 8 occurred. In the online HTML version of the article, Fig. 8 was presented as a duplication of Fig. 7. The original article [1] has been corrected.
