# Supplementary material for: Host-associated microbiomes are predicted by immune system complexity and climate
Source: Genome Biol. 2020 Feb 3;21:23. doi: 10.1186/s13059-019-1908-8 (PMC6996194; doi:10.1186/s13059-019-1908-8)
Supplement: Supplementary file 1 — Additional file 1. Supplementary tables and figures [136–158]. [file 13059_2019_1908_MOESM1_ESM.docx]

**Host-associated microbiomes are predicted by**

**immune system complexity and climate**

**Douglas C. Woodhams, Molly C. Bletz, C. Guiherme Becker, Hayden A. Bender, Daniel Buitrago-Rosas, Hannah Diebboll, Roger Huynh, Patrick J. Kearns, Jordan Kueneman, Emmi Kurosawa, Brandon C. LaBumbard, Casandra Lyons, Kerry McNally, Klaus Schliep, Nachiket Shankar, Amanda G. Tokash-Peters, Miguel Vences, Ross Whetsone**

**Supplementary Materials**

**Table of Contents**

**Datasets.** Provided as separate files.

**Table S1.** Alpha diversity generalized linear models results

**Table S2.** Beta diversity model results

**Table S3.** Immune complexity information

**Figure S1.** PCoA plots of full dataset

**Figure S2.** Alpha diversity metrics across latitude

**Figure S3.** Abundance of major bacterial phyla for external microbiomes

**Figure S4.** Principal coordinates analysis of unweighted Unifrac distances of internal microbiomes of healthy humans from four countries

**Figure S5.** NSTI scores for PICRUSt across host classes

**Figure S6.** Heatmap of predicted functions for internal microbiomes

**Figure S7.** Principal coordinates analysis based on weighted Unifrac distances for digestive microbiomes of insects size-scaled by *Wolbachia* abundance

**Figure S8.** Heatmap of *Wolbachia* sOTU abundance

**Datasets**

Provided as separate files.

**Additional file 2.xlxs** - Full metadata file (15,790 samples), unfiltered

**Additional file 3.xlxs** - Filtered metadata file for internal microbiomes (741 samples)

**Additional file 4.xlxs** - Filtered metadata file for external microbiomes (1193 samples)

**Additional file 5.xlxs** - Filtered metadata file for marine external microbiomes (266 samples)

**Additional file 6.biom** - Deblur sOTU table (biom file) for the full dataset, not rarefied

**Additional file 7.biom** - Deblur sOTU table (biom file) for the internal microbiome (741 samples), rarefied to 1000 reads/sample

**Additional file 8.biom** - Deblur sOTU table (biom file) for the external microbiomes (1193 samples), rarefied to 1000 reads/sample

**Additional file 9.biom** - Deblur sOTU table (biom file) for the marine external microbiomes (266 samples), rarefied to 1000 reads/sample

**Additional file 10.fasta** – RepSet fasta file for the full dataset

**Additional file 11.fasta** - Fasta file to match OTU number with deblur sequence ID

**Additional file 12.tre** - Phylogenetic tree file used for analyses on data subsets

**Supplemental Tables**

**Table S1.** Alpha diversity generalized linear model selection parameter coefficients (Coeff) and variance inflation factors (VIF) for the most parsimonious models for Internal, External, and Marine external microbiome samples. Model coefficients are displayed for OTU richness (OTU), phylogenetic diversity whole tree (PD Whole Tree). Environmental parameters for Internal and External microbiome samples are Bioclimatic variables based on GPS coordinates of sampling location [104] and Marine parameters are based on ocean climate layers [105] from GPS coordinates of sampling location.

**Table S2.** Results of Adonis2 analysis of factors contributing to microbiome community structure (beta-diversity) based on the weighted Unifrac distance matrices for internal (A), external (C), and marine external (D) bacterial communities. The predicted function of the internal microbiomes (B) is also presented with host class maintaining a large proportion of the explanatory power for both community structure and function of these communities. Significant factors in bold. See text for variable selection method. Note that similar results were obtained with unweighted Unifrac, and Principal Coordinates Analysis plots are presented to illustrate the factors explaining the most variation in community structure for internal and external microbiomes (Fig. 4).


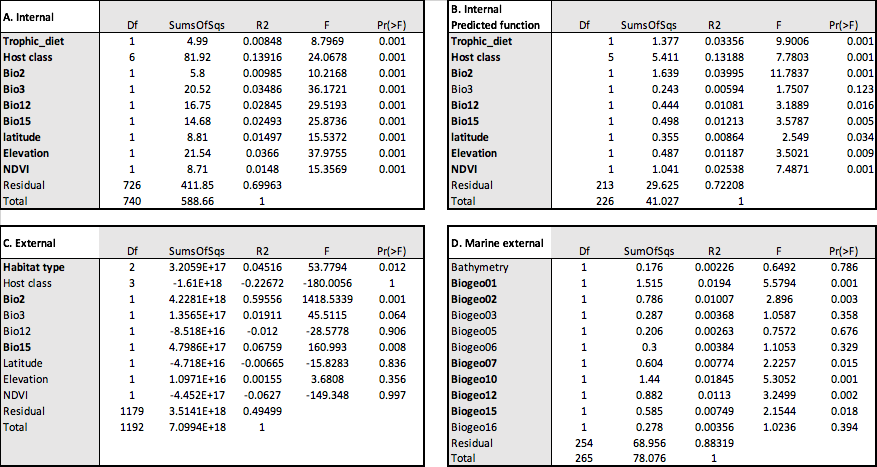


**Table S3.** Internal microbiome sOTU richness for each host class and the corresponding scale ranking adaptive immune complexity based on the adaptive immunity matrix from Flajnik [40]. SHM - somatic hypermutation; CSR - class switch recombination; TCRδ -T cell receptor δ chain; IgVH - immunoglobulin heavy chain variable region; MHC – major histocompatibility complex; DCs - dendritic cells; FDCs - follicular dendritic cells. The mean proportion (averaged across genera) of the microbiome belonging to core sOTUs is also calculated for host classes where possible.

***Preliminary core microbiome analyses****.* A core microbe was defined as an sOTU shared among 80% or more of the samples. Core microbiomes were calculated independently for the internal and external datasets for each host genus with at least five samples. The total sequence reads associated with the core sOTUs for a given sample were then calculated using a custom bash script to allow us to calculate the proportion of the community made up by core sOTUs.

Taxa with greater microbiome diversity tended to have lower abundance of core microbes (Pearson correlation, r = -0.671, N = 7 host classes, 1-tailed P = 0.049). Of the six classes of host animals, insects had the greatest abundance of core microbiota (mean of 16 genera: 38% of reads were associated with core sOTUs), while mammals had the lowest abundance of core microbiota (mean of 22 genera: 22%), and humans were in one of the four mammalian genera that did not have any core bacteria. More rigorous analyses are needed on the relationship between core microbiomes at various specificity (strain level to functional redundancy) among host taxa and in comparison to microbiome diversity or host immune function.


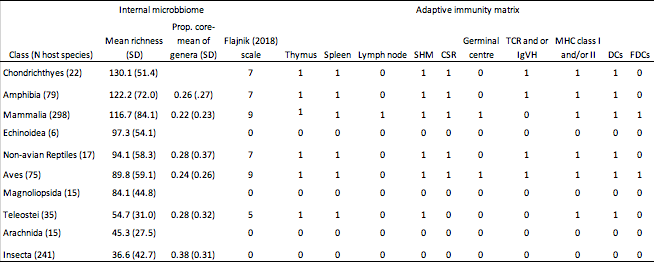


**Supplemental Figures**


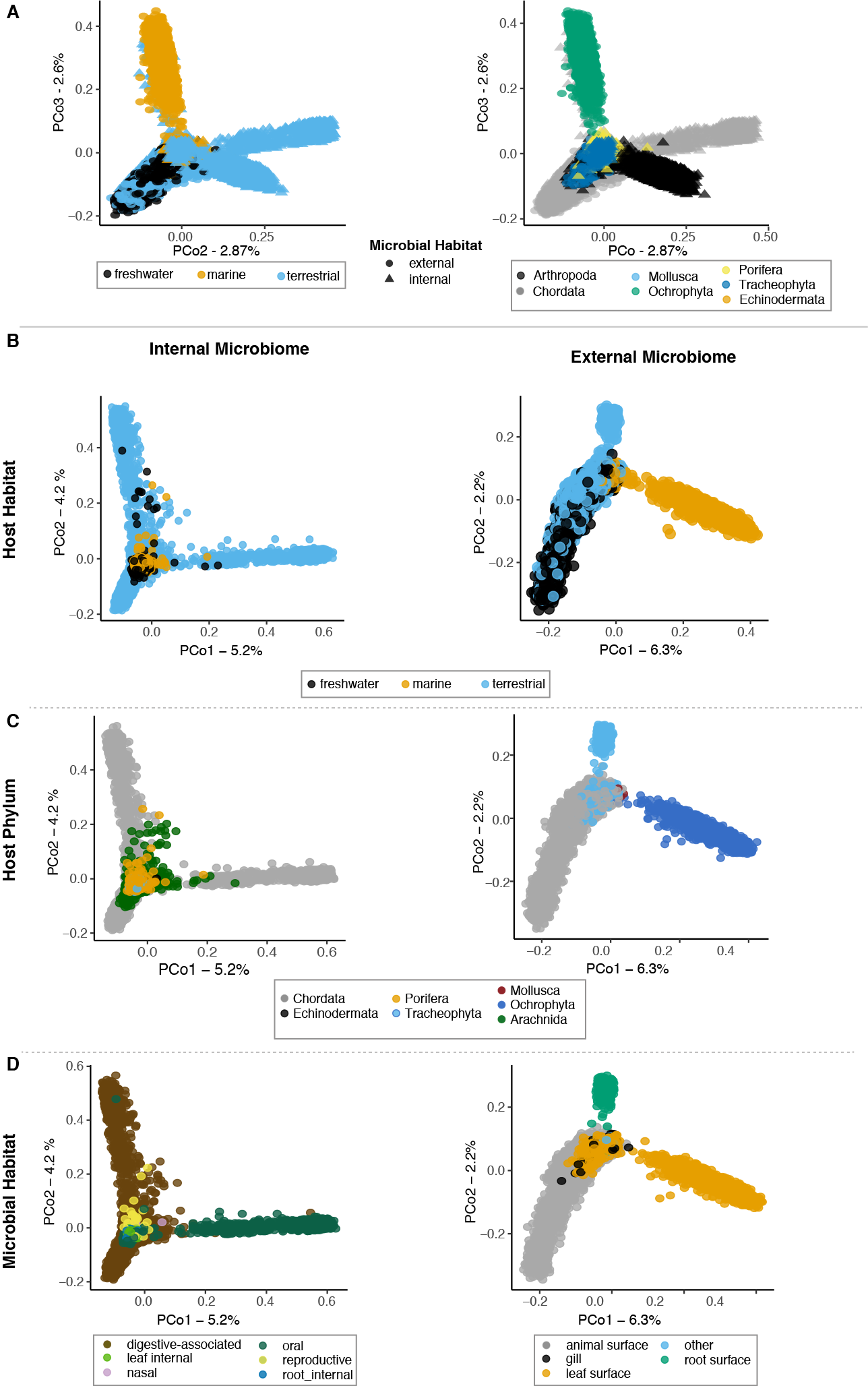


**Figure S1.** Host microbiomes differ across host habitats, host phyla, and microbial habitats. Each plot represents a Principal Coordinates Analysis of the full dataset. (A) Community composition of host microbiomes colored by host habitat (left) and host phylum (right). (B) Community composition of internal (left) and external (right) colored by host habitat. (C) Community composition of internal (left) and external (right) colored by host phylum (D) Community composition of internal (left) and external (right) colored by microbial habitat. Color and shape information is provided below each plot.


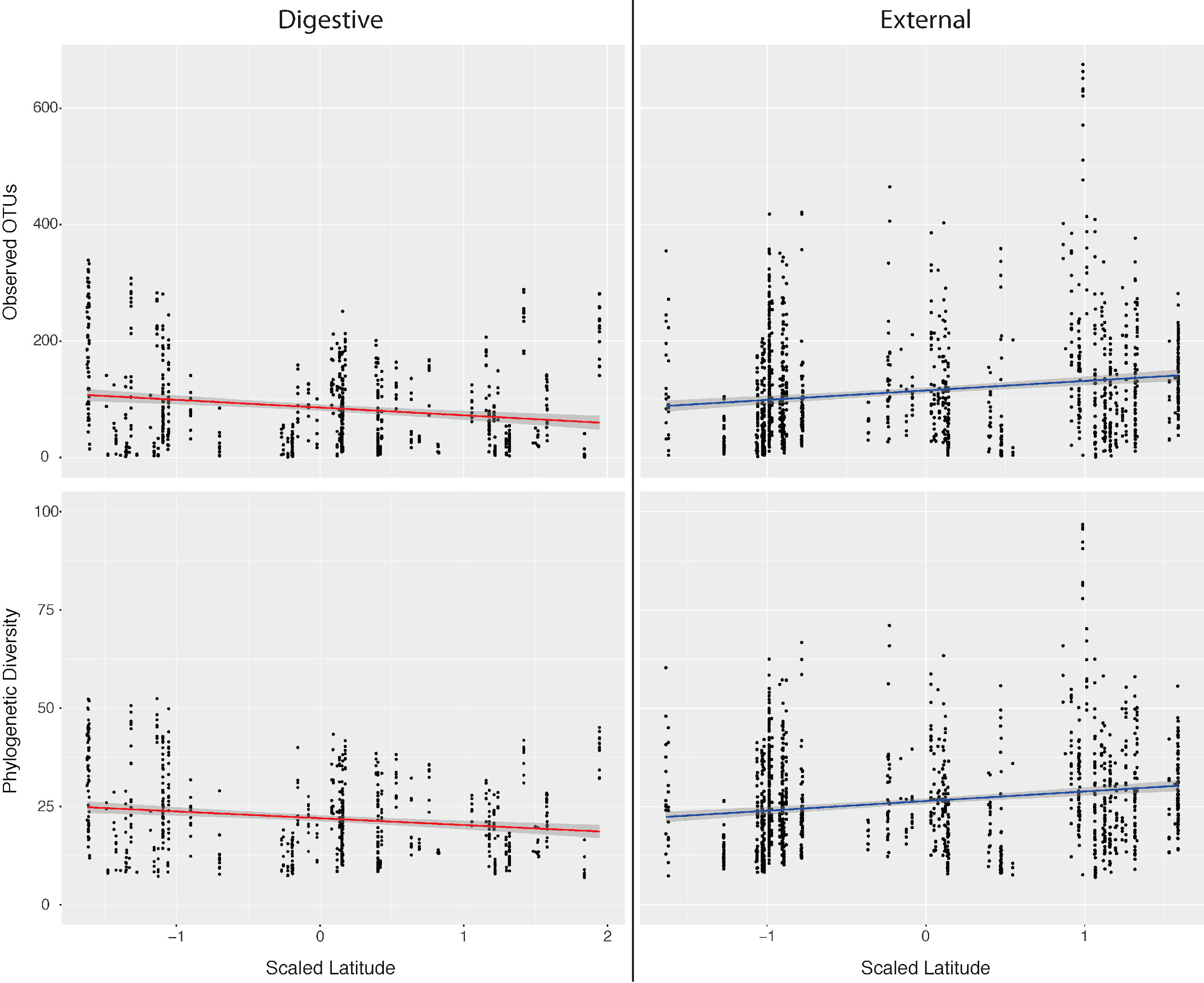


**Figure S2.** Alpha Diversity metrics of studied bacterial communities across latitude. OTU Richness and Phylogenetic Diversity are shown with opposite trends for internal (left panels) or external microbiomes (right panels). The latitudinal-biodiversity gradient weakly followed the macroecological trend for internal microbiomes, but external microbiome diversity increased toward temperate zones.

**
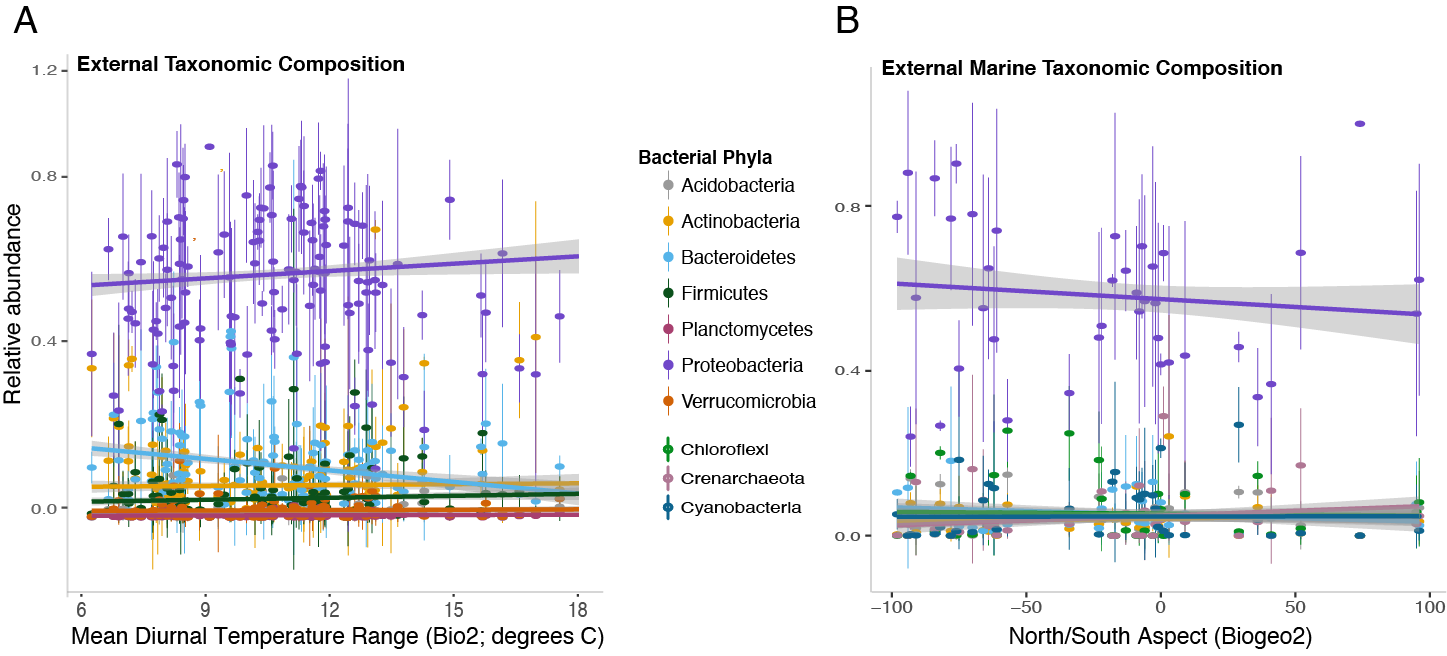
**

**Figure S3.** Abundance of major bacterial phyla for external microbiomes. (A) taxa abundance of external microbiomes of freshwater and terrestrial organisms are displayed across Bio2, and (B) taxa abundance of marine external microbiomes across biogeo2. Bio 2 and Biogeo2 were selected because they were the predictor explaining the most variation in generalized linear models.


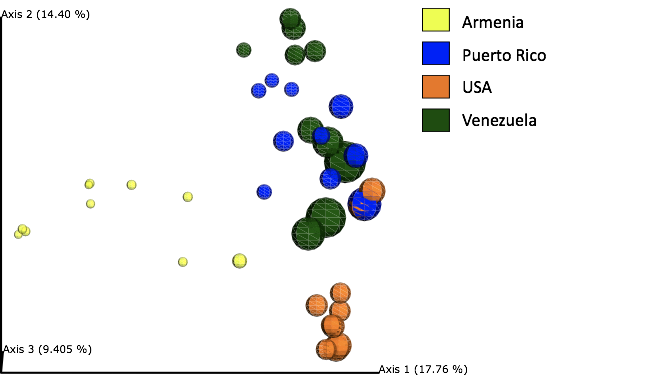


**Figure S4.** Principal coordinates analysis of unweighted Unifrac distances of internal microbiomes of healthy humans from four countries scaled by sOTU richness indicating that subsampling and filtering of our dataset does not eliminate, and recapitulates, previously described patterns [65,71,83,Dominguez-Bello MG - Qiita ID 1774].


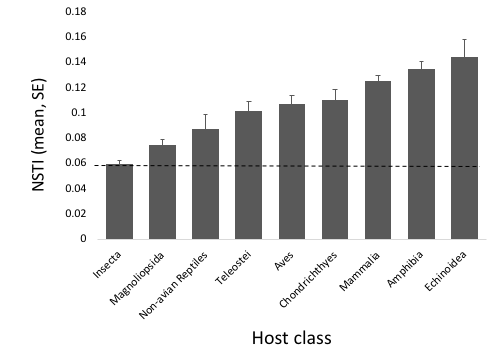


**Figure S5.** The weighted Nearest Sequenced Taxon Index (weighted NSTI) score compared across host classes. NSTI characterizes PICRUSt’s accuracy by calculating the average branch length that separates each bacterial OTU in a sample matched to the GreenGenes database from a reference bacterial genome. NSTI differed significantly among host classes (Kruskal-Wallis test, 𝜒^2^_9_ = 190.36, P < 0.001) For PICRUSt analyses, we filtered the internal microbiome dataset to include only samples where predictive power was high, below the NSTI threshold of 0.06 (dashed line). This reduced the dataset to 247 samples and excluded some taxa (Amphibia, Echinoidea) from further analysis.


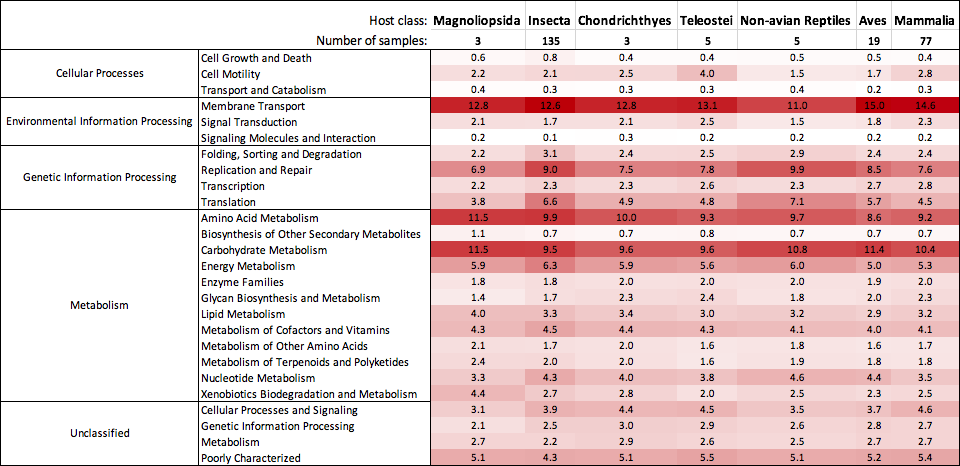


**Figure S6.** Heatmap of predicted functions of internal microbiome based on Level 3 KEGG Ortholog Pathways derived from PICRUSt analysis.


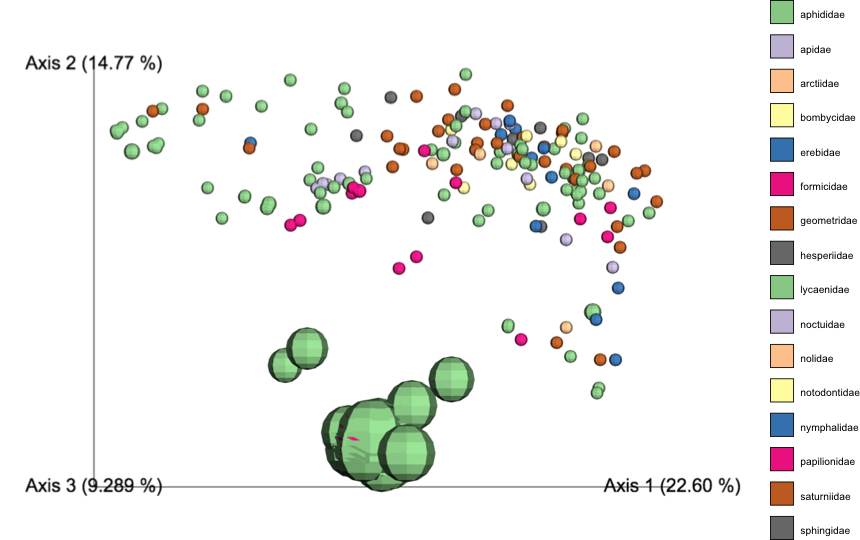


**Figure S7.** Principal coordinates analysis based on weighted Unifrac distances for digestive microbiomes of insects colored by host family, size-scaled by *Wolbachia* abundance. The lycaenidae butterfly communities appear to be dominated by *Wolbachia*. Note however that the abundance of *Wolbachia* can be influenced by the reproductive state of the hosts and is therefore not necessarily comparable across all host taxonomic groups at the time of sampling.


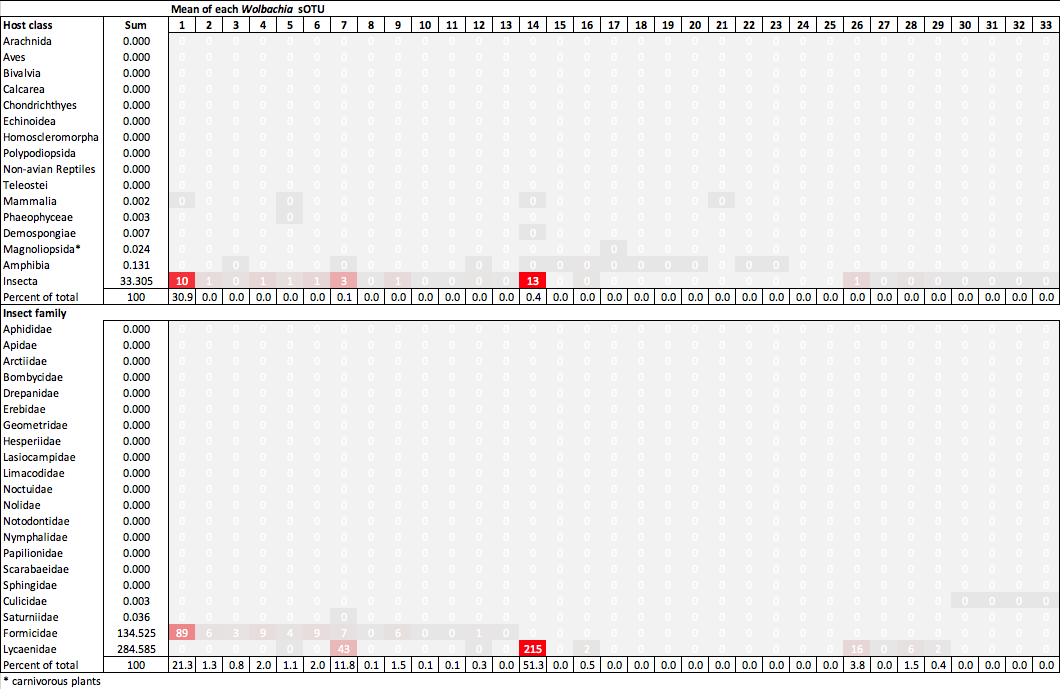


**Figure S8.** Heatmap of *Wolbachia* sOTU abundance. Mean abundance of each of 33 sOTUs in host classes and insect families.

***Wolbachia* in insects are globally diverse and decrease in abundance with temperature range.** *Wolbachia* is a genus of intracellular bacteria with an estimated prevalence of 52% in arthropod species [136–140]. We found it in 21% (33/157 species) of insects in our dataset. Commonly thought of as reproductive parasites that can skew host sex ratios and create cytoplasmic incompatibility, *Wolbachia* may also provide host benefits including pathogen defense [141–143]. Recent advances in our understanding of *Wolbachia* has allowed for the rapid development of novel types of vector control, with a specific focus on mosquitoes [35,92,144,145]. While our understanding of the role of *Wolbachia* has advanced greatly, there are still substantial knowledge gaps in how *Wolbachia* interact with their hosts [35,36,146,147]. For instance, in some mosquito species *Wolbachia* has effectively provided protection from pathogen colonization, while in other species it has aided in the establishment, and ultimately the transmission, of these pathogens [35,144,146,148–150]. Here, we used the internal microbiome subset of our global dataset, which includes whole body samples from many insects, to demonstrate focal use of the data, as well as to scratch the surface on understanding some of the potential global trends affecting *Wolbachia*. Indeed, *Wolbachia* can dominate the microbiomes of certain insects through colonization of the reproductive system, or somatic distribution [151–153], and can subsequently impact microbiome community structure ([92]; Additional file 1: Fig. S7)

The internal microbiome data was analyzed for general trends in *Wolbachia* sOTUs among host classes, and mean abundance within a sample including the majority of samples lacking *Wolbachia* (Table 2). Because several studies have indicated that high temperatures or daily temperature ranges may be associated with reduced abundance of this symbiont [92,154,155], we tested these bioclimatic factors with our dataset. Using a zero-inflated negative binomial distribution we tested a model with the factors maximum temperature of the warmest month, and mean diurnal temperature range (scaled Bioclim 5, and scaled Bioclim2, respectively; °C). These factors predicted relative *Wolbachia* abundance (N=236; Bioclim5: χ² = 10.0614, P = 0.0015; Bioclim2: χ² = 9.1821, P = 0.0024; Fig. 11). A study showing that *Wolbachia*-infected *Drosophila* also thermoregulate by choosing cooler conditions [156] also supports the conclusion *Wolbachia* may be harmed by rising or high temperatures that can be tolerated by some hosts. Further analysis of the 33 *Wolbachia* sOTUs (Table 2) may provide insight on strain-specific variability in host or temperature adaptations [157], and coinfection with *Spiroplasma*, negative co-occurrence with *Asaia*, or other symbionts [143,158]. This is just one example of how this global microbiome dataset can be used to better understand and analyze host-microbe interactions.
