# Supplementary material for: Host-associated microbiomes are predicted by immune system complexity and climate
Source: Genome Biol. 2020 Feb 3;21:23. doi: 10.1186/s13059-019-1908-8 (PMC6996194; doi:10.1186/s13059-019-1908-8)
Supplement: Supplementary file 13 — Additional file 13. Review history. [file 13059_2019_1908_MOESM13_ESM.docx]

Review History

**First round of review**

**Reviewer 1**

**Are you able to assess all statistics in the manuscript, including the appropriateness of statistical tests used?**

Yes, and I have assessed the statistics in my report.

**Comments to author:**

This is an excellent meta analysis of microbiota data across a wide geographic scale, revealing patterns in the internal and external host microbiota with different environmental parameters. The analysis is detailed and thorough, the methods are appropriate, sufficient detail is provided to ensure replication, and the work represents a significant advance that is likely to be of outstanding interest to a broad audience of biologists. My main concern is with a few issues with over interpretation or insufficient statistical justification for particular statements. These are detailed below. I have also identified a few smaller concerns.

1. Core microbiota (L232-240). The authors conclusions that immune complexity trades off with the presence of a core microbiome is fully consistent with the data at hand but, in my opinion, don't reflect a big picture consideration of the data; in particular with respect to sampling breadth. For example, the insecta are represented by just 3 orders, including honeybees that have an exceptionally conserved microbiota (PMID 22558460, 23124740, 22092273, 14615218, 16423014, 18663527). However, they omit other well-studied insects such as Drosophila that are demonstrated to have no core microbiota (PMID 23719154). Thus, the addition or loss of even a few samples might dramatically shift the significance of the detected association. Additionally, no core was detected in humans, which were the most broadly sampled species in the analysis (40 samples). Since a core microbiota is likely to be correlated with sampling breadth within a particular taxonomic level, I suspect these findings may not accurately reflect the biology of the living systems being discussed and I recommend they be removed. I recognize the interpretations are consistent with the data and there could be workarounds - manually adding a few more insecta orders or rarefying each species to a defined set of taxa when defining a core - but I don't think these points are essential to the strength of the manuscript and simply recommend they be removed. Also, an 80% definition of a 'core' microbe may or may not be controversial.

2. Correlation vs. causation for immunity. Immune complexity is correlated with, not causal, for the changes in diversity. The text should be updated to reflect this, including at L69 (driven by -> associated with), L230 ( 'influence on' -> 'association with'), L355 (drivers -> these things were associated with...) lines 396-9, 406-8, 429-32. The authors could potentially address if the proposed paradox of increased complexity correlating with increased diversity could be due to other factors (size of the gut is an obvious one), but this is not necessary for accuracy as long as causation is not overstated.

3. Statements requiring statistical support.

L159 - what is the evidence of underrepresentation? Is there a statistical number that can be attached to this evaluation?

L190-2 the authors should support this statement with a statistical test

L230-2 - since the correlation in 6D is not significant it is not correct to state there is a tradeoff for 6D data with the significant correlation in 6C. This sentence should be revised to be consistent with the data.

Smaller issues:

I recommend to use 'microbiota' for taxonomic or 16S classification and 'microbiome' for shotgun or functional classification.

L167 - I believe the authors should only cite ref 36 here. Also, I am concerned with this statement since is has been clearly shown that the internal and external microbiota are distinct in at least one well-studied insect, Drosophila melanogaster (Cox 2007), and since the cited ref 36 shows a clear clustering of samples based on their surface sterilization; I recognize the p-values are not different, but the clustering is obvious and noted by the authors.

L181-3: 4 points:

- negatively correlated with [internal microbiota diversity]?

- change to reference Fig S2

- please add a correlation test to report if the trend is significant

- were all internal samples digestive?

L212 internal or digestive?

L212-3 why write it this way since everything is significant for internal; whereas not everything is significant for predicted function. I recommend to state clearly hat is thee without the generalization.

L218 - please add a reference for the teleostei

L 267 - please define peripheral

L249-87 - in my opinion this text distracts from the message at hand and could be removed without loss of meaning.

L 289 - 324 - I recommend that this text be moved to the concluding portion of the results section as a 'case study' in implementation.

L292 - I recommend to add several citations and to expand the estimated range: PMID 25772521, 28303186, 25904667

Figure 2 - function -> functional. Is this the right way to present - studies such as Muegge might argue that displaying internal composition by diet, rather than taxonomy, makes more sense...

Figure 4 - please restructure so that 'Diversity' is not cut off

Figure 5 - This figure isn't a very clear way to provide information on 8 bacterial classes. Would it be possible to show a taxon plot broken down by the indicated categories and shading just the 8 classes that display a visual difference? If there is something essential being shown by the current structure, I recommend the authors make this explicit in the text.

reorder Fig S4 to Fig S3 for continuity in the text. Remove or cite in the text Fig s3.

**Reviewer 2**

**Are you able to assess all statistics in the manuscript, including the appropriateness of statistical tests used?**

Yes, and I have assessed the statistics in my report.

**Comments to author:**

In this study, Woodhams et al. explored the association between microbial communities, the host, and their habitats. The authors hypothesized that external host-associated microbiomes responds mostly to global bioclimate or abiotic factors, while internal and digestive system associated microbiomes responds to host factors. While the authors collected and re-analyzed an expressive amount of data, the manuscript is not organized, methods are confusing, figures have very poor resolution (hard to read or not readable), and important information is missing. All the discussion regarding Wolbachia is based on a poor correlation analysis, which calls into question the quality of the approach presented in this study.

MAJOR COMMENTS

The authors state that they used a standard analysis pipeline and cited a review with several recent tools in microbiome analysis, so it is not clear how the results here were obtained. How the data were analyzed in this study should be presented in a very detailed way. Please, provide a flowchart with the tools and the parameters used in this analysis.

Figure Box 1 should be revised. It is hard to read due the poor resolution. Please include in the legend key that it is an Eukaryotic kingdom taxonomy. Chromista is duplicated. It is not clear to me how the tree was obtained. Please revise the methods section to include missing information, such as genetic distance, tree scale. The methods should be re-organized to sub-sections. Figure 1 is hard to read as well, due the poor resolution. How the figure was created should be described in the methods, not in the legend.

Table 1 does not provide the groups information (internal, external, marine external). Which sample belongs to each group? Why did the authors create a separated group for marine samples?

The statement "For insects, surface sterilization does not impact community structure and the microbiomes of whole organism samples may be indistinguishable from the microbiomes sampled from the gut, a body site with consistently high abundance of microbes [36,37]." is incorrect. A study publish by Lanan et al, 2016 (ISME) showed that Cephalotes rohweri has distinct microbial community composition when comparing leg and gut. Although, the microbial load might be very different and in overall, external surfaces might not strongly contribute to the number of OTUs identified, the text has to be modified. Additionally, reference 37 does not contain any information about insect microbiome.

How were bacterial taxa defined? It seems that GreenGenes was used as the reference database (again, the analysis is not clear). However, in Figure 2 amongst all bacterial phyla, "Archea" is presented as taxa. Is it supposed to be the domain Archaea? If yes, Archea is not a bacterial taxa and it is spelled incorrectly.

GLM applied to reduce set of variables was not well explained. How were the least cross-correlated variables selected? Letters in Figure 3 are missing. Legend key indicating the meaning for colors, arrows types (solid, dashed), and arrow tickness scale should be provided. What do all the abbreviations (Bio 3, Bio 14, ...) stand for?

The path analysis assumes that all variables should be linear and independent. However, geophysical factors, such as precipitation and latitude are better explained by polynomial functions. Additionally, temperature, latitude, and precipitation are not independent variables. The authors should provide extra explanation showing that the approach used is acceptable to non-linear and non-independent variable.

Overall, the manuscript is poorly written, the analyses cannot be reproduced due to lack of clear description of methods used and the data is presented very poorly (missing labels, axis, figures not referenced, etc.).

MINOR COMMENT

Please revise all Figures and Table for abbreviation. The meaning should be included in the legend.

Line 187: Figure 3 reference in the text is incorrect or the figure is incomplete. Moreover, I am not sure what the authors want to say in the sentence "internal microbiomes were more diverse toward the tropics", since none of the figure present beta-diversity by latitude. Supplemental Table S2 is confusing to read, the legend "internal (A), external (B)", but in the tables we have "A. Internal, B. Internal Predicted Function, C. External". What is B?

Lines 192-196: "Microbiotas on external surfaces of hosts were more diverse at higher latitudes (Fig. 3) and were structured most strongly by bioclimatic factors (bioclim2 and bioclim15 explaining 60% and 7% of variation, respectively), as well as external habitat type (5%) as compared to host class (not significant; Supplemental Table S2)." Figure 3 does not show the information discussed in the text. Text is very confusing.

The functional composition of the internal microbial communities is extremely similar. How different is the result shown in the manuscript compared with the functional classes available in PICRUSt? Is this maybe a bias introduced due to the sample selection/filtering?

Lines 212-215: Supplemental Table S2 does not show the information discussed in the text.

Line 221: Figure 5B is not discussed in the text.

Line 225: "Ps" is not commonly used for pvalue. Figure 6A y-axis is missing. They should not be presented as figure title.

How was the adaptive immune system complexity defined? By the sum of the boolean classes? How was the core microbiome defined?

Line 233-234: "Taxa with greater microbiome diversity tended to have lower abundance of core microbes (Pearson correlation, r = -0.671, N = 7 host classes, 1-tailed P = 0.049)". Data is not presented in the manuscript.

Figure S7 does not show the information discussed in the text. Please, indicate "humans" in the plot.

Line 233-234 and Figure S7: Why did the authors apply different correlation methods for similar analysis? Negative correlation presented in Figure S7 is not reliable.

Box 2: Top of table, less than one read per sample? What does it mean?

Box 2: The correlation plot is biased by few samples. Samples as clearly distributed in two groups (high and low abundance). The authors should be careful about their conclusions based on correlations analysis like the one presented here. The relevance of the example discussed in the manuscript is not clear.

Line 371-371: Figure reference in the text is incorrect. Fig 2 is Fig 3? Again, the information is not clearly presented.

The discussion regarding Wolbachia is based on a poor correlation analysis. Data discussed in the line 311 and 313 are not shown in the manuscript.

**Authors Response**

**Point-by-point responses to the reviewers’ comments:**

Reviewer #1: This is an excellent meta analysis of microbiota data across a wide geographic scale, revealing patterns in the internal and external host microbiota with different environmental parameters. The analysis is detailed and thorough, the methods are appropriate, sufficient detail is provided to ensure replication, and the work represents a significant advance that is likely to be of outstanding interest to a broad audience of biologists. My main concern is with a few issues with over interpretation or insufficient statistical justification for particular statements. These are detailed below. I have also identified a few smaller concerns.

1. Core microbiota (L232-240). The authors conclusions that immune complexity trades off with the presence of a core microbiome is fully consistent with the data at hand but, in my opinion, don't reflect a big picture consideration of the data; in particular with respect to sampling breadth. For example, the insecta are represented by just 3 orders, including honeybees that have an exceptionally conserved microbiota (PMID 22558460, 23124740, 22092273, 14615218, 16423014, 18663527). However, they omit other well-studied insects such as Drosophila that are demonstrated to have no core microbiota (PMID 23719154). Thus, the addition or loss of even a few samples might dramatically shift the significance of the detected association. Additionally, no core was detected in humans, which were the most broadly sampled species in the analysis (40 samples). Since a core microbiota is likely to be correlated with sampling breadth within a particular taxonomic level, I suspect these findings may not accurately reflect the biology of the living systems being discussed and I recommend they be removed. I recognize the interpretations are consistent with the data and there could be workarounds - manually adding a few more insecta orders or rarefying each species to a defined set of taxa when defining a core - but I don't think these points are essential to the strength of the manuscript and simply recommend they be removed. Also, an 80% definition of a 'core' microbe may or may not be controversial.

*We are glad the reviewer thinks that our conclusions are appropriate for the data we analyzed. We are aware that while the number of species examined in this study is large, it does not include all groups, even some well-studied groups like Drosophila, because the available studies did not use sequencing methods consistent with our analytical approach, or were not from wild populations. Thus, data could not always be combined, and many studies were not included. Instead of adding additional species to this analysis in an ad hoc fashion, we have chosen to remove this analysis from the paper. This should not affect the overall conclusions of the paper, and we may be better able to explore this topic in a future meta-analysis. We have thus removed the previous Figure 6D from the paper, as suggested by the reviewer.*

*Note that we have retained supplementary tables and figures that include core analysis, now termed “preliminary” in Supplemental Table S3, and carefully worded a short discussion of our hypothesized trade-off between core components of the microbiome and immune complexity in the supplement.*

2. Correlation vs. causation for immunity. Immune complexity is correlated with, not causal, for the changes in diversity. The text should be updated to reflect this, including at L69 (driven by -> associated with),

*Wording changed, as suggested.*

L230 ( 'influence on' -> 'association with'),

*Wording changed, as suggested.*

L355 (drivers -> these things were associated with...) lines 396-9, 406-8, 429-32.

*Wording changed, as suggested.*

The authors could potentially address if the proposed paradox of increased complexity correlating with increased diversity could be due to other factors (size of the gut is an obvious one), but this is not necessary for accuracy as long as causation is not overstated.

*We have made the requested revisions to the text to be more cautious.*

3. Statements requiring statistical support.

L159 - what is the evidence of underrepresentation? Is there a statistical number that can be attached to this evaluation?

*Here we visually inspected the global map (Fig. 1) and noted that few samples were included in our analysis from Africa and Asia. Thus, they are underrepresented geographic areas in our dataset.*

L190-2 the authors should support this statement with a statistical test

*We have provided PERMANOVA tests in Supplemental Material Table S2 and richness data in Table S3. These stats are now referenced in the text, as suggested.*

L230-2 - since the correlation in 6D is not significant it is not correct to state there is a tradeoff for 6D data with the significant correlation in 6C. This sentence should be revised to be consistent with the data.

*The analyses of core microbiomes were removed and we also removed figure 6D.*

Smaller issues:

I recommend to use 'microbiota' for taxonomic or 16S classification and 'microbiome' for shotgun or functional classification.

*While we respect that different definitions and descriptors are used for 16S rRNA targeted gene studies, we prefer to continue using accepted terminology to match published studies and ongoing projects including the Earth Microbiome Project and Human Microbiome project that use the term “Microbiome”. Our study draws heavily on the Earth Microbiome Project (Thompson et al. 2018, Nature).*

L167 - I believe the authors should only cite ref 36 here. Also, I am concerned with this statement since it has been clearly shown that the internal and external microbiota are distinct in at least one well-studied insect, Drosophila melanogaster (Cox 2007), and since the cited ref 36 shows a clear clustering of samples based on their surface sterilization; I recognize the p-values are not different, but the clustering is obvious and noted by the authors.

*We agree with the reviewer that internal and external insect microbiomes differ. As described above, we have clarified our intended meaning in this section, and also in the new Figure 1 of the methodological flow diagram. We include reference 37 - Sender R, Fuchs S, Milo R. Revised estimates for the number of human and bacteria cells in the body. PLoS Biol. 2016;14:1–14. – because it demonstrates the large biomass of microbial cells in the vertebrate gut comparing organs such as the large intestine and skin surface (their Table1). We have revised this section for clarity.*

L181-3: 4 points:

- negatively correlated with [internal microbiota diversity]?

- change to reference Fig S2

- please add a correlation test to report if the trend is significant

- were all internal samples digestive?

*Changed, as suggested. Correlation results are described in Supplemental Table S1, now referenced in the text. Internal samples are described in the methods section and include some whole-body samples.*

L212 internal or digestive?

*Changed to “internal” for consistency.*

L212-3 why write it this way since everything is significant for internal; whereas not everything is significant for predicted function. I recommend to state clearly hat is thee without the generalization.

*Internal microbiomes and functional capacities were structured by similar variables (only Bioclim3 was not significant for function). Our hypotheses stated in the introduction was that function may differentiate host species more strongly than microbiomes, but this was not the case. The two measures were equivalent. We have revised this section for clarity.*

L218 - please add a reference for the Teleostei

*We added these references:*

*Xiao Joe JT, Chiou PP, Kuo CY, Jia Lin JH, Wu JL, Lu MW. 2019. The microbiota profile and transcriptome analysis of immune response during metamorphosis stages in orange spotted grouper (Epinephelus coioides).*

*Fish Shellfish Immunol. 2019 Jul;90:141-149. doi: 10.1016/j.fsi.2019.03.063.*

*Egerton S, Culloty S, Whooley J, Stanton C, Ross RP. 2018.*

*The Gut Microbiota of Marine Fish. Front Microbiol. 2018 May 4;9:873. doi: 10.3389/fmicb.2018.00873.*

*Yan Q, Li J, Yu Y, Wang J, He Z, Van Nostrand JD, Kempher ML, Wu L, Wang Y, Liao L, Li X, Wu S, Ni J, Wang C, Zhou J. 2016. Environmental filtering decreases with fish development for the assembly of gut microbiota. Environ Microbiol. 2016 Dec;18(12):4739-4754. doi: 10.1111/1462-2920.13365.*

L 267 - please define peripheral

*Definitions were added for core and peripheral.*

L249-87 - in my opinion this text distracts from the message at hand and could be removed without loss of meaning.

*This text is meant to be a stand-alone section (Box 1) and was moved to the end of the manuscript.*

L 289 - 324 - I recommend that this text be moved to the concluding portion of the results section as a 'case study' in implementation.

*This is another stand-alone section (Box 2) and has been moved to the end of the manuscript. It is, as the reviewer suggested, a case study.*

L292 - I recommend to add several citations and to expand the estimated range: PMID 25772521, 28303186, 25904667

*Since the cited reference is from 2012, we have now added the suggested citations, plus an additional up-to-date review (2019):*

*Sazama EJ, Ouellette SP, Wesner JS. 2019. Bacterial Endosymbionts Are Common Among, but not Necessarily Within, Insect Species. Environ Entomol. 2019 Feb 13;48(1):127-133. doi: 10.1093/ee/nvy188.*

*de Oliveira CD, Gonçalves DS, Baton LA, Shimabukuro PH, Carvalho FD, Moreira LA. 2015. Broader prevalence of Wolbachia in insects including potential human disease vectors. Bull Entomol Res. 2015 Jun;105(3):305-15. doi: 10.1017/S0007485315000085.*

*Sazama EJ, Bosch MJ, Shouldis CS, Ouellette SP, Wesner JS. 2017. Incidence of Wolbachia in aquatic insects. Ecol Evol. 2017 Jan 24;7(4):1165-1169. doi: 10.1002/ece3.2742.*

*Weinert LA, Araujo-Jnr EV, Ahmed MZ, Welch JJ. 2015. The incidence of bacterial endosymbionts in terrestrial arthropods. Proc Biol Sci. 2015 May 22;282(1807):20150249. doi: 10.1098/rspb.2015.0249.*

Figure 2 - function -> functional. Is this the right way to present - studies such as Muegge might argue that displaying internal composition by diet, rather than taxonomy, makes more sense...

*Figure 2B has now been removed. Microbiome function data is presented in the text and supplement.*

Figure 4 - please restructure so that 'Diversity' is not cut off

*Revised, as suggested.*

Figure 5 - This figure isn't a very clear way to provide information on 8 bacterial classes. Would it be possible to show a taxon plot broken down by the indicated categories and shading just the 8 classes that display a visual difference? If there is something essential being shown by the current structure, I recommend the authors make this explicit in the text.

*We decided to retain the information about phylogenetic relatedness in all groups (part A), and then focus on the 8 bacterial classes (part B) showing which groups are and which groups are not differing with trophic diet. We have revised the text to clarify the main message.*

reorder Fig S4 to Fig S3 for continuity in the text. Remove or cite in the text Fig s3.

*Supplementary Figure S3 has now been referenced in the text.*

Reviewer #2: In this study, Woodhams et al. explored the association between microbial communities, the host, and their habitats. The authors hypothesized that external host-associated microbiomes responds mostly to global bioclimate or abiotic factors, while internal and digestive system associated microbiomes responds to host factors. While the authors collected and re-analyzed an expressive amount of data, the manuscript is not organized, methods are confusing, figures have very poor resolution (hard to read or not readable), and important information is missing. All the discussion regarding Wolbachia is based on a poor correlation analysis, which calls into question the quality of the approach presented in this study.

*We have now revised the manuscript for clarity and organization, included a new flow chart (Figure 1A) to inform the reader of our methods which we clearly describe in the Methods section at the end of the paper. We reformatted figures for clarity. We removed a “core” analysis figure and moved discussion of core primarily to the supplement. We moved the Box 1 and Box 2 stand-alone sections to the end of the manuscript. We have also removed the Wolbachia correlation and chosen a more appropriate analysis for this box, which is intended to provide an example of how the data set can be utilized for specific microbial taxa of interest to better understand the microbial ecology.*

MAJOR COMMENTS

The authors state that they used a standard analysis pipeline and cited a review with several recent tools in microbiome analysis, so it is not clear how the results here were obtained. How the data were analyzed in this study should be presented in a very detailed way. Please, provide a flowchart with the tools and the parameters used in this analysis.

*We now provide a flowchart of our methodology and have updated the methods for detail and clarity. All of the analyses presented are reproducible with the descriptions in the methods section and the supplemental data files. We now clearly reference these files in the text.*

Figure Box 1 should be revised. It is hard to read due the poor resolution. Please include in the legend key that it is an Eukaryotic kingdom taxonomy. Chromista is duplicated. It is not clear to me how the tree was obtained. Please revise the methods section to include missing information, such as genetic distance, tree scale. The methods should be re-organized to sub-sections.

*We have provided the methods for the Eukaryotic tree in the legend key, and increased font size and resolution for readability. The tree was retrieved from TimeTree (http://www.timetree.org), and a link for readers to interact with the tree is now included in the legend. The tree aggregates taxonomic and phylogenetic information from published literature. The purpose of the tree is less about accuracy in detailed phylogenetic relationships, and more of a tool to present information about hosts that have been sampled for microbiomes in order to indicate missing groups. We think this is a good way to organize this information visually. The methods have several sub-sections and appear as the last main section in the Genome Biology article.*

Figure 1 is hard to read as well, due the poor resolution. How the figure was created should be described in the methods, not in the legend.

*High resolution files are now provided, and methods clarified.*

Table 1 does not provide the groups information (internal, external, marine external). Which sample belongs to each group? Why did the authors create a separated group for marine samples?

*Table 1 has been updated and does provide information for internal, external and marine external. In the text and Fig. 1 diagram, we have further explained why marine external is separate from terrestrial/aquatic external. Based on the Earth Microbiome Project ontogeny, marine samples are distinct. In addition to comparing external samples from different habitats (marine vs. terrestrial/aquatic), we wanted to compare internal and external samples from the same habitat, thus included only terrestrial/aquatic samples for this comparison. The dataset did not have adequate samples from internal marine samples to include the fourth category.*

The statement "For insects, surface sterilization does not impact community structure and the microbiomes of whole organism samples may be indistinguishable from the microbiomes sampled from the gut, a body site with consistently high abundance of microbes [36,37]." is incorrect. A study publish by Lanan et al, 2016 (ISME) showed that Cephalotes rohweri has distinct microbial community composition when comparing leg and gut. Although, the microbial load might be very different and in overall, external surfaces might not strongly contribute to the number of OTUs identified, the text has to be modified. Additionally, reference 37 does not contain any information about insect microbiome.

*We have carefully revised this section as both Referees point out that internal and external microbiomes differ in insects, a fact that is not in dispute in this study. Rather, our intention was to explain how we classified the whole-organism samples that are presented in some studies of insects. Whole-organism insect samples were classified as internal. This classification for each sample is included in the metadata provided in supplementary materials, and we now also include in the methods flow chart Figure 1A - a depiction of how the samples were analyzed.*

How were bacterial taxa defined? It seems that GreenGenes was used as the reference database (again, the analysis is not clear).

*We refer the Referee to our detailed Methods section, and our new flow chart figure, as suggested for clarity.*

However, in Figure 2 amongst all bacterial phyla, "Archea" is presented as taxa. Is it supposed to be the domain Archaea? If yes, Archea is not a bacterial taxa and it is spelled incorrectly.

*This has been corrected, thanks for detecting the error.*

GLM applied to reduce set of variables was not well explained. How were the least cross-correlated variables selected?

*We refer the Referee to our detailed Methods section, and our new flow chart figure, as suggested for clarity.*

Letters in Figure 3 are missing. Legend key indicating the meaning for colors, arrows types (solid, dashed), and arrow thickness scale should be provided. What do all the abbreviations (Bio 3, Bio 14, ...) stand for?

*An arrow thickness scale is now provided in the revised figure. Bioclimatic variables are explained in the text, and also described in the Supplemental Table S1. We have added the definitions to the figure legends as appropriate. Figure legends appear at the end of the manuscript text, but will appear with the figure upon publication. It is pasted here:*

*Figure 3. Path analyses showing direct and indirect effects of the best abiotic and biotic predictors of phylogenetic diversity and number of sOTUs. Models explaining internal (a, b) and external microbiome diversity (c, d) are shown. Numbers are standardized path coefficients (*P < 0.05). Blue arrows depict positive associations whereas red arrows depict negative effects. Grey arrows depict non-significant paths. The thickness of the arrows represents the relative strength of each relationship. Bioclimatic variables include: Isothermality (Bio3), Mean Temperature of Driest Quarter (Bio9), Precipitation of Driest Month (Bio14), and Precipitation of Warmest Quarter (Bio18).*

The path analysis assumes that all variables should be linear and independent. However, geophysical factors, such as precipitation and latitude are better explained by polynomial functions. Additionally, temperature, latitude, and precipitation are not independent variables. The authors should provide extra explanation showing that the approach used is acceptable to non-linear and non-independent variable.

*We fully addressed the reviewer's comment by removing latitude from the four path models explaining internal and external microbiomes (Fig. 3) because of high multicollinearity when including several indirect effects of latitude on bacterial diversity; removing latitude resolved the problem. We provide revised path models, and have correspondingly changed our results section. Latitudinal effects are likely mediated by bioclimatic variables, and we discuss these factors in relationship to both internal and external microbiomes.*

Overall, the manuscript is poorly written, the analyses cannot be reproduced due to lack of clear description of methods used and the data is presented very poorly (missing labels, axis, figures not referenced, etc.).

*We believe, as described above, that our methods are reproducible following our careful methods section, the new flow chart in Fig. 1A, and the data files provided in Supplementary Materials. We have revised the text for clarity following reviewer suggestions. Figures have been updated and Figure legends appear at the end of the manuscript after References.*

MINOR COMMENT

Please revise all Figures and Table for abbreviation. The meaning should be included in the legend.

*We have now checked the figure legends and include all abbreviations in the legends.*

Line 187: Figure 3 reference in the text is incorrect or the figure is incomplete. Moreover, I am not sure what the authors want to say in the sentence "internal microbiomes were more diverse toward the tropics", since none of the figure present beta-diversity by latitude.

*In our previous Fig. 3, the red arrow from latitude to phylogenetic diversity or number of OTUs indicated a significant negative correlation with latitude. Alternatively, for external microbiomes, the blue arrows from latitude to phylogenetic diversity or number of OTUs indicated positive correlations.*

*We have now removed latitude from these path models (Fig. 3) and have thus revised our discussion to indicate that latitudinal effects on internal and external microbiomes are likely mediated by bioclimatic variables.*

Supplemental Table S2 is confusing to read, the legend "internal (A), external (B)", but in the tables we have "A. Internal, B. Internal Predicted Function, C. External". What is B?

*Fig. 2B was removed. The legend has been revised.*

Lines 192-196: "Microbiotas on external surfaces of hosts were more diverse at higher latitudes (Fig. 3) and were structured most strongly by bioclimatic factors (bioclim2 and bioclim15 explaining 60% and 7% of variation, respectively), as well as external habitat type (5%) as compared to host class (not significant; Supplemental Table S2)." Figure 3 does not show the information discussed in the text. Text is very confusing.

*We agree that the text needed clarification, and we have revised it according to our revised figures.*

The functional composition of the internal microbial communities is extremely similar. How different is the result shown in the manuscript compared with the functional classes available in PICRUSt? Is this maybe a bias introduced due to the sample selection/filtering?

*As also suggested by reviewer 1, we have removed the PICRUST functional comparison among hosts figure (formerly Fig. 2B). Functional results are found in Supplementary Table S2.*

Lines 212-215: Supplemental Table S2 does not show the information discussed in the text.

*We found an error in the legend that may have cause this confusion. Supplemental Table S2 does have both internal microbiome (panel A) and the PICRUST functional characterization of the internal microbiome (panel B).*

Line 221: Figure 5B is not discussed in the text.

*We now reference Fig. 5B in the text: Shifts in major classes with four bacterial phyla are illustrated in Figure 5B.*

Line 225: "Ps" is not commonly used for pvalue.

*This has been corrected.*

Figure 6A y-axis is missing. They should not be presented as figure title.

How was the adaptive immune system complexity defined? By the sum of the boolean classes? How was the core microbiome defined?

*This figure has been modified to include the y-axes labels rather than headers. We have removed the core analysis figure as suggested by Reviewer 1, and the path analysis has been promoted to its own Figure 7. Adaptive immune system complexity is defined in the text (see Methods section) and also in Supplemental Table S3.*

Line 233-234: "Taxa with greater microbiome diversity tended to have lower abundance of core microbes (Pearson correlation, r = -0.671, N = 7 host classes, 1-tailed P = 0.049)". Data is not presented in the manuscript.

*This section was removed according to suggestion from Reviewer 1, and a separate paragraph “Preliminary core microbiome analyses” under Supplementary Table S3 describes these results and also shows the data used.*

Figure S7 does not show the information discussed in the text. Please, indicate "humans" in the plot.

*The humans are plotted as one of 4 points at zero core for the mammals, but the figure has been removed, see below.*

Line 233-234 and Figure S7: Why did the authors apply different correlation methods for similar analysis? Negative correlation presented in Figure S7 is not reliable.

*We agree that the correlations described statistically in Supplemental Figure S7 are not obvious visually, and we have decided to remove this analysis and figure. The core data were removed from the main text upon suggestion from Reviewer 1, and do not add significantly to the story we are focused on here. The data will be better analyzed in a separate manuscript focused on core microbiomes.*

Box 2: Top of table, less than one read per sample? What does it mean?

*This has now been better described in the text. Because there are many samples with zero Wolbachia, the mean is less than one per sample.*

Box 2: The correlation plot is biased by few samples. Samples as clearly distributed in two groups (high and low abundance). The authors should be careful about their conclusions based on correlations analysis like the one presented here. The relevance of the example discussed in the manuscript is not clear.

*This case study in Box 2 has been moved to the end of the manuscript, and is an example of using the dataset to examine ecological hypotheses such as high temperature effects on Wolbachia abundance among insect hosts. We agree that the data were not analyzed appropriately, and have revised this analysis to include a model with a zero-inflated negative binomial distribution, as the reviewer described. The result is the same. There are significant relationships, and Wolbachia abundance is lower at high temperatures (or rather, scaled Bioclim5 and Bioclim2 as described). A new figure replaces the old.*

Line 371-371: Figure reference in the text is incorrect. Fig 2 is Fig 3? Again, the information is not clearly presented.

*Thank you for the correction. Fig. 3 is now correctly referenced. We have now combed the manuscript to ensure that the correct figures and tables are referenced, and the legends are clear.*

The discussion regarding Wolbachia is based on a poor correlation analysis. Data discussed in the line 311 and 313 are not shown in the manuscript.

*We have replaced this analysis and now show the data for both of the Bioclims hypothesized to affect Wolbachia abundance.*

*We thank the reviewers for their careful reading of the manuscript leading to several important improvements.*

**Second round of review**

**Reviewer 1**

The authors addressed my concerns.

I believe references at L173-4 the references here are not correct. I believe 173 should be 38 and 174 should be 39. The authors should carefully check all references. All surrounding references are appropriate.
